# Recent Development in Molecular Dynamics Simulations of Gas Hydrates in Flow Assurance

**DOI:** 10.1021/acs.energyfuels.5c00558

**Published:** 2025-05-19

**Authors:** Parisa Naeiji, Mengdi Pan, Judith M. Schicks, Niall J. English

**Affiliations:** † School of Chemical and Bioprocess Engineering, 8797 University College Dublin, Belfield, Dublin 4, Ireland; ‡ GFZ Helmholtz Centre for Geosciences, Telegrafenberg, 14473 Potsdam, Germany

## Abstract

The efficient production of oil and gas is paramount to meet global energy demands, necessitating a focus on flow assurance in pipelines. Gas hydrate formation, along with other issues, like corrosion and deposition, poses significant threats to fluid transportation systems in pipelines, leading to significant economic and safety risks. Various operational and chemical strategies have been developed to mitigate hydrate formation in oil and gas pipelines, but challenges remain in terms of cost-effectiveness and environmental impact. Molecular dynamics (MD) simulations offer a promising avenue for understanding hydrate behavior and designing effective inhibition strategies. This review consolidates current knowledge on MD simulations of gas hydrates, focusing on their application in flow assurance. By examination of recent advancements and challenges, this review aims to foster innovative strategies and technologies for ensuring the reliability, safety, and sustainability of hydrocarbon transportation systems in gas hydrate-prone environments.

## Graphical abstract

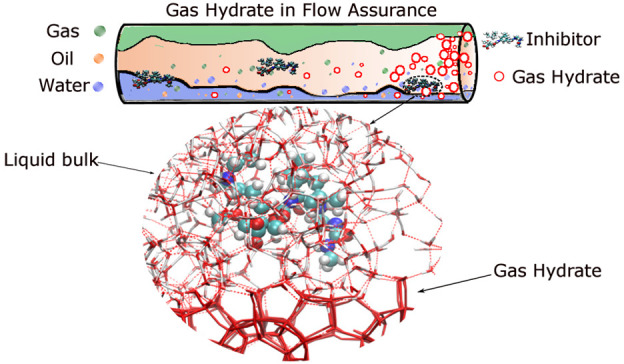

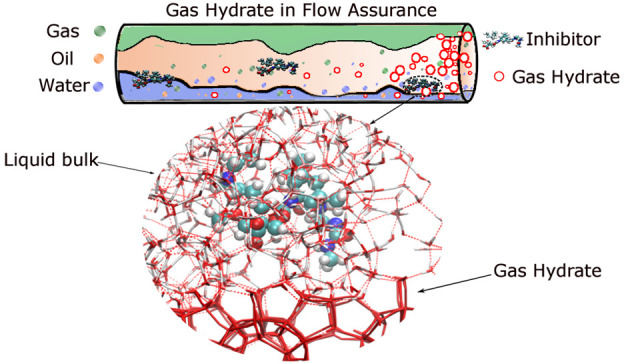

## 1 Introduction

Gas hydrate formation, pipeline corrosion, wax deposition, scale deposition, asphaltene precipitation, and carboxylate fouling pose great threats when transporting hydrocarbon fluids from offshore wellheads to production and processing platforms.^1,2^ Among them, gas hydrate formation has been regarded as the leading source of flow assurance problems since the first discovery of hydrate blockages in pipelines in 1934.^3–5^ The unanticipated formation of gas hydrates in pipelines may lead to substantial economic consequences and critical safety risks.

Gas hydrates are ice-like crystalline structures formed by hydrogen-bonded water molecules that create a three-dimensional lattice, capable of trapping small gas molecules such as CH_4_, CO_2_, and other suitably sized hydrocarbons.^6^ The occurrence of gas hydrates is restricted to the areas where low-temperature and high-pressure conditions prevail. Depending on the size of the enclosed guest molecules, hydrates with cubic structure I (sI), structure II (sII), and hexagonal structure H (sH) have been verified from samples retrieved from natural reservoirs.^7–9^ Small guest molecules like CH_4_, C_2_H_6_, and CO_2_ typically form sI hydrates, while larger hydrocarbons tend to form sII hydrates. Even bulkier molecules, such as neo-hexane, can stabilize sH hydrates when accompanied by a smaller helper gas (e.g., CH_4_) occupying the smaller cavities.^10^ The substantial quantities of hydrocarbon gases encapsulated in hydrate structures, along with their widespread natural distribution, have sparked significant interest in recent decades as a promising alternative energy resource.^8,11,12^

Despite their potential as an energy resource, gas hydrates pose a significant challenge to flow assurance in the oil and gas industry. Under favorable conditions, they can readily form, agglomerate, and accumulate, leading to flow blockages, operational disruptions, and safety risks, especially in subsea pipelines and production systems.^3,13,14^ Hydrate blockages can occur at various stages of hydrocarbon handling, including production, processing, and transportation.^15,16^ With the presence of enough water and hydrocarbon gases (hydrate-forming gases) in the pipelines and the suitable thermodynamic conditions prevailing there, gas hydrates can easily form. As hydrate formation increases, the solid–liquid ratio in the hydrate slurry rises, potentially resulting in either a sudden pipeline blockage or the gradual development of a hydrate film along the pipe wall.^17^ Furthermore, the turbulent flow and impurities which act as crystallization centers may accelerate the hydrate formation process.^18^ Figure 1 depicts a conceptual model for hydrate blockage in non-emulsifying systems involving initial phase separation stage, the hydrate growth and deposition stage, and the agglomeration and bedding of hydrates which eventually lead to the buildup of hydrates and plugging of the pipelines.^19,20^ Consequently, understanding the behavior of gas hydrates in terms of nucleation and growth, as well as developing effective strategies for their management has become urgent issues to tackle the problem.

**Figure 1:**
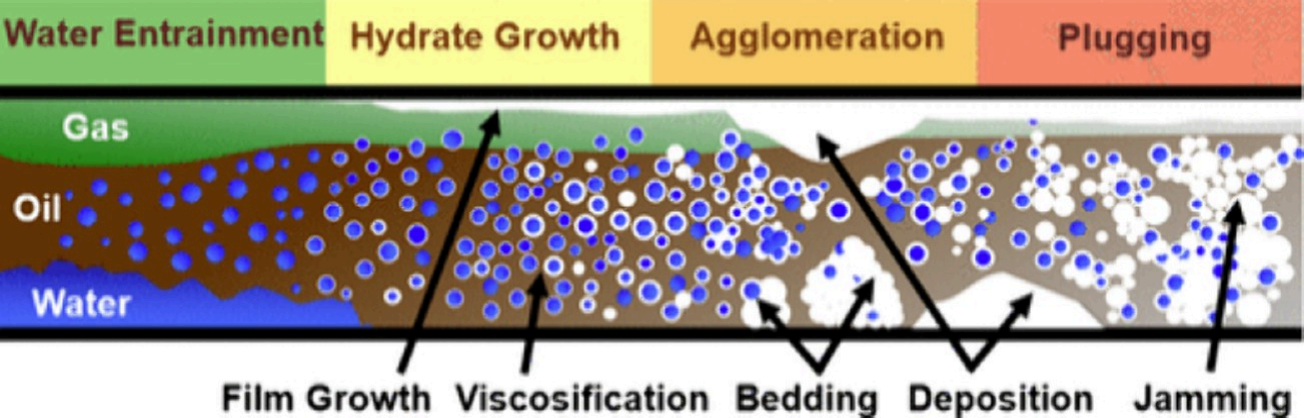
Conceptual model for gas hydrate formation in non-emulsifying systems. This figure was reproduced with permission from ref 20. Copyright 2020 American Chemical Society.

In recent years, the oil and gas industry has developed and implemented various methods to manage hydrate risks, including both operational or physical approaches and chemical strategies. On the one hand, the system’s thermodynamic operation conditions (basically temperature and pressure) are controlled in order not to reach the hydrate stability zone. Operational strategies such as depressurization, heating, and dehydration are used to mitigate hydrate formation.^13^ An alternative approach involves injecting chemical inhibitors into pipelines. These include thermodynamic hydrate inhibitors (THIs), which shift the hydrate equilibrium conditions; kinetic hydrate inhibitors (KHIs), which delay the initial formation and slow the growth of hydrate crystals; and anti-agglomerants (AAs), which prevent the clumping of already-formed hydrate particles, allowing them to remain dispersed and flow freely.^15,21^ However, none of these methods are ideal in consideration of the economical and environmental perspective.^21^ Alternative forms of green and high-efficiency strategies are still urgently needed.

Even though laboratory work has been intensively carried out for the study of gas hydrate properties^22–25^ and the assessment of inhibition strategies,^26–29^ experimental approaches remain limited in their ability to capture nanoscale processes across both spatial and temporal dimensions. To overcome these challenges, computational approaches, particularly molecular simulations, have emerged as valuable tools for studying gas hydrate formation. Bearing this in mind, this review aims to consolidate the current state of knowledge in molecular dynamics (MD) simulations of natural gas hydrates with a specific focus on their application in the realm of flow assurance.

There is a limited number of studies that provide a comprehensive review of gas hydrate properties through MD simulation, as well as relevant research focused on simulating gas hydrates with a flow assurance perspective.^14,21,30,31^ However, most of these studies take a broad approach, encompassing both experimental and molecular simulation aspects, with less emphasis on MD studies and the underlying mechanisms of the processes involved. Additionally, some reviews focus on only one particular method of investigating gas hydrates in flow assurance, such as inhibiting their formation through chemical injection, while neglecting other important areas. Moreover, there are very few review papers that address risk assessment and physical strategies for inhibiting gas hydrates. Therefore, the novelty of this review paper lies in that it provides an extensive overview of the MD simulation studies, including risk assessment and minimization, operation strategies for gas hydrate inhibition, more effective chemical strategies of both thermodynamic and kinetic inhibitors, and anti-agglomerants. By elucidating the recent advancements and challenges on this topic, we hope to contribute to the development of innovative strategies and technologies that enhance the reliability, safety, and sustainability of hydrocarbon transportation systems in a gas hydrate-prone environment within the oil and gas industry.

## 2 MD Simulation of Gas Hydrate in Flow Assurance

MD simulations have become an essential tool for exploring the intricate molecular-level mechanisms of natural gas hydrate nucleation, crystal growth, dissociation, and phase behavior under varying conditions.^32–36^ MD simulations offer an opportunity to explore the dynamic interactions between gas molecules and water molecules, providing a valuable perspective into the structural, thermodynamic, and kinetic aspects of gas hydrates, aiding in the formulation of strategies to mitigate their formation and ensure the continuous flow in the oil and gas pipelines.^31^

While MD simulations provide detailed molecular- and atomic-level insights, their limitations in time and size scales can hinder direct comparisons with experimental results. Experiments typically observe phenomena over longer time scales and larger systems than those currently accessible to MD simulations, making validation against experimental data challenging. Furthermore, it is challenging to provide an accurate MD simulation of a complex system. Gas hydrate nucleation in multicomponent systems is a widespread phenomenon in nature and poses a variety of scientific and industrial challenges. At lower temperatures, gas hydrates typically nucleate in the aqueous phase near the gas–liquid interface, where the process is largely governed by mass transfer. As the temperature increases, the preferred nucleation site shifts gradually from the gas–liquid interface to the solid (silica surface)–liquid interface. Under this condition, the nucleation free energy barrier plays a dominant role in the formation process, making heterogeneous nucleation, characterized by a lower free energy barrier, more favorable.^37^ Moreover, the selected temperature regulation method may significantly impact the dynamics and characteristics of hydrate formation. The origin of this effect remains an open question.^38^ Beyond the above-mentioned challenges, other critical factors, such as the selection of appropriate force fields for molecules and the development of more robust force fields for multicomponent systems or transferability of parameters developed for bulk systems to interfacial simulations, also significantly impact the accuracy of MD simulations, as discussed later in this section. These challenges can be mitigated by carefully reviewing relevant literature and conducting trial MD simulations, allowing researchers to assess and refine the accuracy of their results.

Despite these limitations, MD simulations remain invaluable in scientific research, particularly in gas hydrate studies and flow assurance, due to their unique capabilities. MD simulations enable researchers to study the nucleation, growth, and dissociation of gas hydrates at the molecular scale, providing insights that are difficult to capture experimentally. This knowledge is crucial for developing strategies to control hydrate formation and improve flow assurance in pipelines. In addition, MD simulations enable the evaluation of various inhibitors’ effectiveness in preventing hydrate formation, aiding in the development of more efficient and cost-effective chemical inhibitors.

Prior to discussing specific works on hydrate molecular simulation *per se* as applied to flow-assurance phenomena, and the broader context of flow-assurance strategies themselves which can benefit from molecular-simulation insights, it is useful, perhaps, to consider the appropriateness of molecular simulation methods in and of themselves in gas-hydrate molecular simulation. In this context, the application of ideal (classical, pairwise) potential models to hydrate systems has been a longstanding challenge, especially in kinetics studies where a thorough understanding of the underlying phase diagram is required to determine the “driving forces” for nucleation, growth, and other processes.^39–41^ However, even for accurately calculating the subtle details of equilibrium and transport properties, such as thermal conductivity, using more “tailored” potential models becomes essential, especially in relation to host–guest interactions.^42–45^ Actually, a variety of research has used a diversity of possible models (e.g., different guest–host combining rules, rigid and flexible, fixed-charge and polarizable, for both water and guests, etc.) to highlight the variation in the outcomes.^45–48^ There have been relatively few efforts so far in hydrate simulations to use interaction parameters and potential models for water and guest molecules that have been specifically parametrized for water-guest or hydrate systems. Similarly, it could be argued that for CH_4_ hydrate, the original TIP4P water model and an ab initio-fitted five-site CH_4_ model^49^ might be ideal for specific characteristics (like melting point^43,49^ ), but for phase equilibrium,^50^ MP2-fitted model is also remarkable (along with argon hydrate). The MP2-fitted model of Velaga et al.^51,52^ is arguably superior to the Harris–Yung CO_2_ potential model^53^ for CO_2_ hydrate, and English and Clarke’s recent studies on CO_2_ hydrate dissociation^54^ found also good melting point estimation. To be sure, Jiang et al. and English and MacElroy^47,55,56^ have shown that polarizable water models, the “charge-on-spring” and AMOEBA models, generally provide quantitatively better results for the treatment of (CH_4_) hydrates.^55^ But even with “state-of-the-art” water models, careful configuration of guest–host interactions is still necessary to get the most accurate results. There has not been much use of polarizable potential models in hydrate simulation,^44,47,48,56,57^ however this might be significant in external electric or e/m fields.^45,56^ The single-particle tetrahedral bias water potential (and its CH_4_ counterpart) by Jacobson et al. enables faster sampling of kinetics, both due to the model’s design and its accelerated kinetic response.^41^

Even considering the applicability of classical MD *per se* (as opposed to path-integral approaches), when simulating at low temperatures (below about 100–150 K), MD’s approximation to classical dynamics may be in doubt; this is especially the case for hydrogen hydrates. It is advised to use path-integral MD (PIMD) in this situation; a more thorough examination of PIMD’s requirements is outside the purview of this review. Conde et al.^39^ used PIMD to simulate hydrates and observed that this method works well below 150 K. It might be time to question the assumption that empirical pairwise models “trained” for (ambient temperature) classical MD can be seamlessly applied to PIMD with the expectation of achieving more accurate results. Cendagorta et al. also used PIMD to calculate the free energy profiles for H_2_ and D_2_ diffusion through clathrate hydrates across temperatures from 8 to 200 K. Their results revealed that the shape of the quantum free energy profiles, as well as their relative height compared to classical profiles, varies significantly with temperature, owing to the intricate balance between competing quantum effects.^58^ To accurately capture the specific mechanisms driving the enhancement of each interaction based on its underlying physical or chemical origin, as well as the representative mechanisms common in hydrogen-containing systems, including electrostatic and van der Waals interactions, a detailed description of these weak molecular interactions necessitates high-level theoretical approaches, such as the use of PIMD.^59^

Aside from potential models and classical or quantum sampling in simulations, over the past 20 years or so, we may gauge these methods by their performance in estimating relative free energies, as a basic check of molecular simulation methods prior to use in flow-assurance simulations. Indeed, in this regard, there has been a growing focus on calculating the free-energy differences between various forms of hydrate, often employing MD-TI methods,^60–63^ despite the use of Monte Carlo (MC) for hydrate stability in slit pores by Chakraborty and Gelb.^64^ Free-energy techniques, however, hold the greatest potential for improving our knowledge of nucleation. An important development in hydrate simulation was the study of CO_2_ hydrates by Radhakrishnan and Trout,^65^ where the Landau free-energy hypersurface was analyzed using various order parameters. It is evident that free-energy approaches have a lot to offer when evaluating current or prospective hydrate inhibitors, and molecular simulation can be a key component of this process as a perfect, predictive “prototyping” design tool. In this context, the MD simulations by Storr et al. both in 2004 ^66^ and Anderson et al.^67^ open up exciting new avenues for the application of free-energy techniques to advance our understanding of hydrate inhibition.

Of course, a vitally important point in molecular simulations applied to the long time scales of flow-assurance lies in the relative ability to sample longer time scales in MD simulations, as also mentioned in the first paragraphs of this section. In recent years, the hydrate-simulation community has made progress toward semiroutinely reaching (near-)­microsecond time scales, e.g. for investigations into nucleation,^68^ CO_2_–CH_4_ replacement mechanisms,^69^ or using “coarse-grained” potentials that permit longer timesteps and do not involve electrostatics.^70^ However, the use of dedicated hardware acceleration for MD simulations of hydrates has also been reported, particularly on the FPGA-based MD-GRAPE 2 ^62,71^ and platforms utilizing graphics processing units (GPUs).^71,72^ Sakamaki et al.^72^ implemented mixed-precision GPU MD for structure H hydrates with minimal accuracy loss, whereas Varini et al.^71^ have employed NVIDIA GPU cards “native” double precision. Varini et al.^71^ provided a helpful exercise that also examined the performance of MD-GRAPE and GPUs for MD acceleration as a function of system size. Specifically, they focused on the computational implementation and parallelization of three-dimensional FFTs for reciprocal space, considering that atomistic models with explicit electrostatics were utilized.

However, beyond the obvious point of the desirability of performing longer molecular simulations, there is also the equally vital point about the fidelity of the flow-assurance modeling by way of reaching adequately large system sizes. Currently, “massively parallel” MD implementations on high-performance supercomputers are essential to simulate very large systems (containing up to millions of molecules), which are necessary to prevent periodic artifacts, particularly in interfacial simulations, over extended time scales (typically on the order of a hundred nanoseconds or a fraction of a microsecond). There has been very little published in this area of hydrate simulation, with typical time scales of over ten years before simulations using “leadership” supercomputing resources become more widely accessible on smaller Beowulf clusters or multiprocessor workstations. That being said, English^73^ has published the results of MD simulations of CH_4_-hydrate systems with up to several million molecules using Blue Gene resources. CH_4_ and water were represented using both fully atomistic and coarse-grained approaches, along with single-particle, no-electrostatics,^41^ an aspect of the latter. In summary, Blue Gene/Q was found to provide fast internode communication and an efficient massively parallel MD at the moment, even for explicit electrostatics.

Of course, the proceeding critique in the present section regarding molecular-simulation methods as applied to hydrate molecular simulation has not yet considered the pros and cons of *ab initio* atomistic simulation. In the vein, density functional theory (DFT), has been used more and more in recent years to perform harmonic calculations and geometry optimization on individual hydrate cages. Due to the O­(N^3^) scaling of the DFT algorithms used, along with dynamic memory limitations, computational efficiency “requires” that the “cage-only” approach focus on the periodic environment of the clathrate, which presents a significant drawback. When dispersion interactions are addressed in some DFT studies, whether they are of individual aperiodic cages,^74–78^ at least, they are addressed explicitly^79,80^ for full periodic hydrate unit cells.^81^ This affords an extra layer of complexity and a possible avenue for future research and development in DFT-level simulation.^82,83^ Naturally, selecting a basis set and addressing any related basis set superposition error (BSSE) through counterpoise correction is a crucial part of DFT simulation, as it significantly impacts the precision of all computations. Some DFT treatments of hydrate cages have taken this into consideration, but Chattaraj et al.^78^ especially have looked at a variety of distinct basis sets, e.g., 3-21G, 6-31G­(d), and 6-31Gp­(d) applied to cages in H_2_ hydrates, and systematically evaluated the energy differences. They also took into account a range of comparable basis sets for MP2, coming to the conclusion that MP2 energy terms were more trustworthy. In order to generate PES for subsequent pairwise forcing, MP2-level simulation has been reported to provide an improved method of treating dispersion in aperiodic guest-water systems.^78^

The introductory discussion on gas hydrate MD-simulations has strengthened our decision to explore MD-simulation studies further, focusing on those that demonstrate their impact on flow assurance challenges. This review examines recent progress in MD simulations of gas hydrates, particularly in relation to flow assurance for oil and gas pipelines. In the risk assessment section, we examined the risk of hydrate blockage and its susceptibility to factors such as coexistence with substances like oil (wax, and asphaltene) as well as the influence of pipeline surface conditions on hydrate formation. In the subsequent section, we reviewed methods for mitigating this risk, encompassing both physical and chemical strategies. This review included the latest developments from 2010 to the present, ensuring a comprehensive understanding of contemporary approaches to hydrate management within flow assurance strategies.

## 3 Risk Assessment

How to quantitatively assess the risk of hydrate blockage based on the specific working condition is a hot topic. During multiphase transportation (water, oil, and gas) through pipelines, the deposition of hydrates and specific oil components, such as wax and asphaltene, often leads to pipeline blockages, becoming a significant flow assurance challenge in the oil and gas industry. If these solids coexist and interact with each other in the arctic or subsea flow system, the risk of blockage or plugging increases, but their interaction is uncertain because they are often investigated separately. This section reviews a quantitative evaluation method for assessing the risk of hydrate blockage through MD simulations, as well as exploring the role of oils in hydrate formation in both bulk water and at the water–gas interface. This knowledge contributes to a more realistic understanding of oil–gas flow assurance challenges when they coexist in a multiphase system.

### 3.1 Precipitation of Gas Hydrate

The process of hydrate formation in pipelines unfolds in three stages: (I) nucleation, growth, and agglomeration of hydrates, (II) hydrate deposition, and (III) hydrate plugging.^84–88^ Surfaces, particularly pipeline walls, play crucial roles in all stages by providing sites for hydrate formation. Pipeline walls, being the coldest component in the system, facilitate hydrate nucleation, growth, and adhesion.^89,90^ Preventing gas hydrate blockages requires reducing hydrate adhesion on pipeline surfaces.

Given the sometimes conflicting experimental and simulation results, it is important to highlight that determining whether hydrates can directly nucleate and grow on solid surfaces remains a challenging task.^91,92^ However, in almost all studies involving solid surfaces, a hydrate nucleus typically forms a stable conglomerate by creating an intermediate layer (referred to as IML) or by interacting with specific functional groups present on the solid surfaces.^93–95^ This microscopic tendency forms the physical basis for hydrate deposition. A notable example is the tendency of hydrate particles to accumulate on pipeline walls during fluid flow or shutdown/restart scenarios, as the hydrate nucleus grows and aggregates.

Ma et al. investigated hydrate adhesion on solid surfaces, with a particular focus on the atomistic structure of the intermediate layer and how it influences the adhesion behavior. They found that the intermediate layer’s structure is a competitive equilibrium regulated by guest molecule content. Both water structure density and guest molecule adsorption determine adhesion strength. Their analysis revealed significant differences in adhesion between ice and hydrate, with ice exhibiting approximately five times greater adhesion strength. Additionally, the study indicates that surfaces exhibiting hydrophobicity and the ability to template low-density water structuring are more effective in minimizing hydrate adhesion.^96^ The findings revealed that the presence of gas molecules at the interface significantly reduces hydrate adhesion strength, with a pure gas layer yielding the lowest adhesion among studied systems. This finding aligns well with experimental results.^97^

While factors such as the thermal stability of natural gas hydrates and surface adhesion are crucial for oil and gas pipeline safety, the influence of pipe surface roughness and hydrophobicity on hydrate stability remains uncertain. To address this, Wu et al.^98^ conducted MD simulations on 12 molecular models of solid steel pipeline surfaces with random morphology. Their study aimed to clarify the kinetics of CH_4_ hydrate dissociation, the nucleation and growth of gas bubbles during hydrate decomposition, and the free energy associated with hydrate adhesion to solid steel surfaces. Their study revealed that increasing the hydrophobicity of the pipe surface by 52% could reduce CH_4_ hydrate thermal stability by up to 85%. The study also observed a shift in the location of gas bubble nucleation, from the bulk water to the solid surface, as surface hydrophobicity increased. However, highly hydrophobic surfaces hindered gas bubble formation on both smooth and rough surfaces. Additionally, the study revealed that the free energy of hydrate adhesion is influenced by both surface roughness and hydrophobicity, with the highest energy barrier observed on hydrophobic surfaces exhibiting high roughness.^98^ These findings provide valuable insights into CH_4_ hydrate evolution concerning changes in pipe wall surface properties due to natural events or artificial treatments.

In pipelines, local temperatures and pressures are governed by fluid dynamics, prompting the critical question of how much water can be present in the gas before condensation, or “drop out”, takes place. Traditionally, this is evaluated using the water dew point. When the actual mole fraction of water exceeds the dew point mole fraction, water condenses locally. If temperature and pressure conditions are favorable, hydrate formation can then occur from the condensed liquid water and hydrate-forming components in the gas phase. Kvamme and Aromada examined Troll gas transportation conditions from the Kollsnes gas processing plant to the continent, focusing on temperature and pressure factors. They investigated the critical issue of water content in the gas phase, comparing traditional water dew point calculations to water adsorption on hematite (Fe_2_O_3_), a component of rust. Their findings showed that water adsorption on hematite dominated over dew-point calculations, suggesting its importance in hydrate nucleation. The water content tolerance based on the dew point can be up to 20 times greater than the water content related to adsorption from gas onto solid hematite surfaces. Given that the average chemical potential of adsorbed water is approximately 3.4 kJ/mol lower than that of liquid water, this adsorption-driven pathway becomes the dominant mechanism in governing the risk of water uptake from the gas phase and, consequently, hydrate formation.^99^ However, earlier MD simulation results by Kvamme et al. suggested that hematite particle surfaces might function as thermodynamic hydrate inhibitors. This is because the chemical potential of water adsorbed on the hematite surface was found to be lower than that in water clusters. The estimated energetic advantage of water adsorbing onto the hematite surface, rather than condensing into droplets, ranged from −1.7 and −3.4 kJ mol^–1^.^100^ Recent MD simulations of Zhang et al. highlighted that in water-dominant systems, the role of the water film differs on iron (Fe) and its corrosion surfaces (Fe_2_O_3_, FeO, and Fe_3_O_4_) compared to gas-dominant systems. On Fe surfaces with strong water affinity, deposited hydrates are unable to transform the adsorbed water into additional hydrate, leading to the formation of a persistent water film. As water affinity decreases across the sequence Fe > Fe_2_O_3_ > FeO > Fe_3_O_4_, the behavior of adsorbed water changes, converting into amorphous hydrate on Fe_2_O_3_ and forming ordered hydrate structures on FeO and Fe_3_O_4_ following hydrate deposition. The strength of hydrate adhesion correspondingly increases with decreasing water affinity, as the absence of a liquid layer makes hydrate detachment more difficult. In contrast to gas-dominant environments, the presence of a water film in water-rich systems actually weakens hydrate adhesion. These insights clarify the mechanisms of hydrate deposition on Fe and its corrosion products, and suggest that modifying surface water affinities could offer a viable strategy for managing hydrate deposition in pipelines.^101^ Another research revealed that carbon steel (CS) corrosion promotes CH_4_ hydrate nucleation by improving gas–liquid interfacial mass transfer. The introduction of a corroded CS coupon into a sodium dodecyl sulfate (SDS) solution has been shown to reduce CH_4_ hydrate induction time by 37%, while also increasing the percentage of water converted to hydrate by 25%. Corroded CS further accelerates hydrate nucleation by 60% due to localized CH_4_ enrichment in corrosion grooves, unlike rust from chemical corrosion, which has minimal effects. Nucleation, mainly at the gas–liquid interface, is dominated by CS corrosion, with other regions contributing insignificantly. Both pristine and corroded CS surfaces influence hydrate formation kinetics primarily during the early nucleation stage, promoting water conversion to hydrate and reducing the hydrate dissociation rate at elevated temperatures. As a result, gas hydrate plugging may become more pronounced in pipelines with corroded surfaces over time, especially at the contact line of the gas–liquid–metal three-phase interface.^102^

### 3.2 Co-precipitation of Gas Hydrate and Asphaltene

Asphaltenes are the densest and most surface-active fraction of crude oil, prone to aggregation due to various interactions, including acid–base interactions, hydrogen bonding, metal coordination complexes, hydrophobic forces, and π–π stacking interactions.^103^ Zi et al. applied the MD simulations to (1) assess the impact of asphaltenes on the overall and local tendencies of hydrate formation in both bulk water and at the water–gas interface, (2) identify the mechanisms behind the observed phenomena by quantifying the partitioning of water and CH_4_, as well as the distribution of different types of hydrate cavities, and (3) evaluate the role of asphaltenes in hydrate decomposition and, in turn, examine how hydrate decomposition influences the segregation of asphaltene aggregates.^104^

Asphaltenes have been found to exhibit both promoting and inhibiting effects on gas hydrate formation. Zi et al. showed that the asphaltenes located at the water–gas interface promote the hydrate formation, primarily due to enhanced CH_4_ diffusion and the facilitated transition from face-saturated incomplete cavities to complete cages. In contrast, asphaltenes in bulk water slightly inhibit hydrate formation. This inhibition is linked to the adsorption of CH_4_ on asphaltene aggregates and the hydrate cavities near asphaltenes, which prevents CH_4_ from being trapped in water cavities. In both scenarios, the presence of asphaltenes increased hydrate crystallization, indicating higher risks of hydrate deposition in the presence of asphaltenes.^104^ The process of CH_4_ hydrate formation and decomposition, along with asphaltene aggregation, is conceptually illustrated in Figure 2.

**Figure 2:**
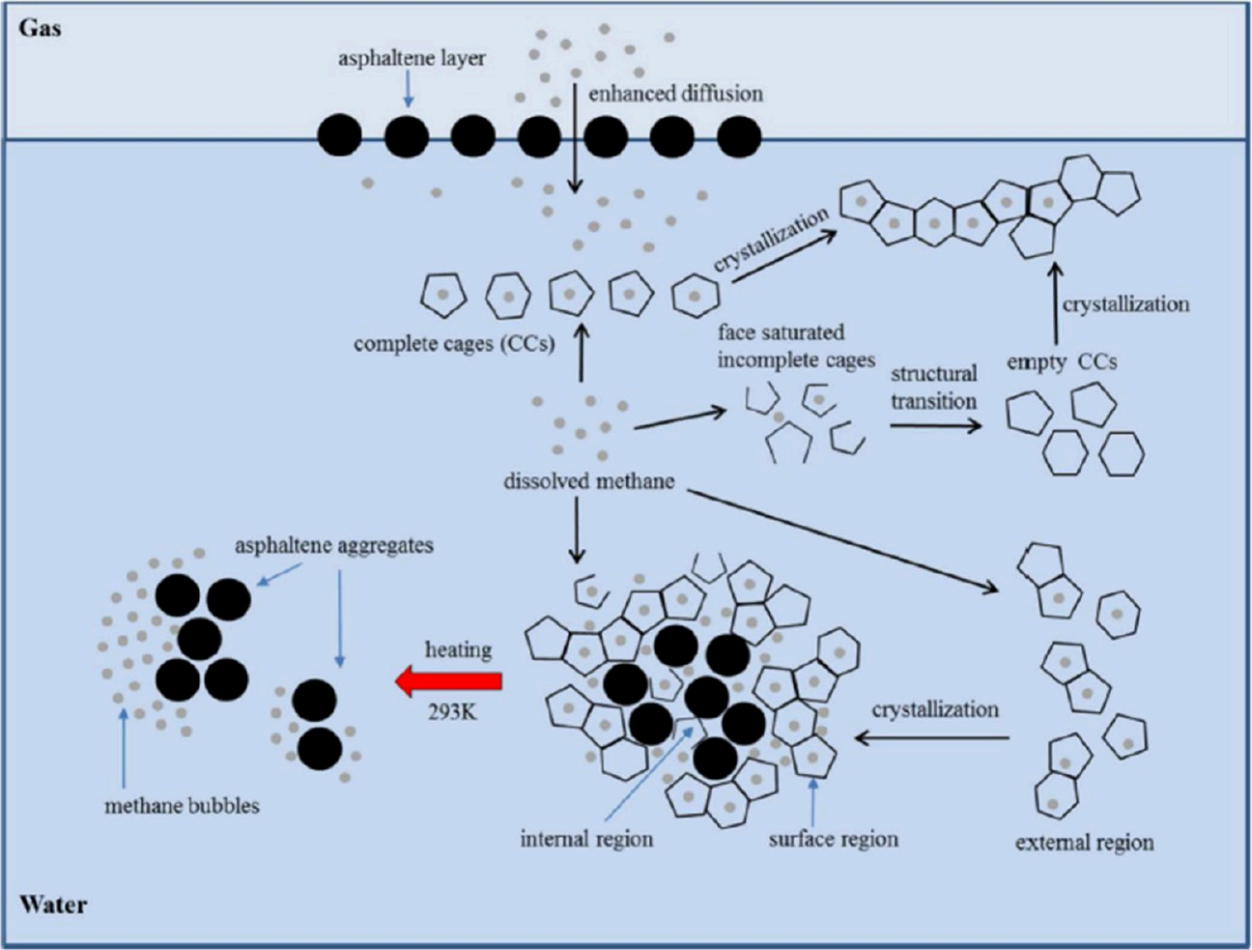
Conceptualization the process of hydrate formation and decomposition in the gas–water–asphaltene system. This figure was reproduced with permission from ref 104. Copyright 2016 American Chemical Society.

Zi et al. investigated the combined impacts of solvent type, water droplet size, and asphaltenes on CH_4_ hydrate formation in a water-in-oil emulsion model.^105^ The MD simulation results offered theoretical insights into the mechanisms behind CH_4_ hydrate formation in asphaltene-rich water-in-oil emulsions. The findings revealed that asphaltenes inhibited CH_4_ hydrate formation, with the effect of being more pronounced in smaller droplets with *n*-heptane or larger droplets with toluene (see Figure 3). This inhibition was attributed to two primary processes closely linked to the surface concentration of asphaltene at the oil–water interface: (1) the formation of an asphaltene shell that prevents CH_4_ dissolution, and (2) the disruption of local hydrogen bonding networks due to hydrogen bonds formed between asphaltene and water.^105^

**Figure 3:**
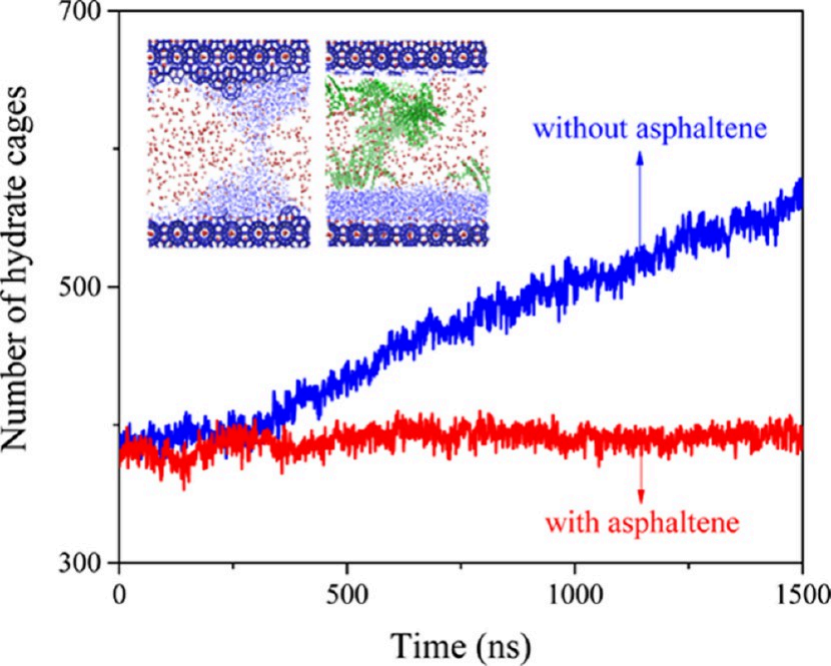
Number of aqueous CH_4_ molecules during the hydrate formation in the systems with/without asphaltene. This figure was reproduced with permission from ref 105. Copyright 2018 American Chemical Society.

In another study, Zi et al. investigated CH_4_ hydrate formation on smooth and rough metal surfaces coated with water, light oil, and heavy oil containing asphaltenes. This study was likely the first molecular-level analysis of CH_4_ hydrate evolution on metal surfaces with heavy oils.^106^ They examined the preferred sites for hydrate formation on pipe walls with concave and convex surfaces, which could result from metal corrosion, changes in pipe diameter, or the deposition of solid particles during multiphase transportation. The study observed that light oil inhibited CH_4_ hydrate growth, with this effect being further enhanced by the addition of asphaltenes. It was also found that hydrate formation favored the grooves of rough metal surfaces, where the hydrate grew upward from the groove and extended to the gas–water interface. The inhibitory effect of asphaltenes on hydrate formation on metal surfaces was influenced not only by the concentration (ranging from 6.7 to 8.3 wt %) but also by the distribution of asphaltenes, particularly at the water–gas interface.^106^

### 3.3 Co-precipitation of Gas Hydrate and Wax

In addition to asphaltenes, the simultaneous presence of wax and gas hydrate deposits in subsea pipelines increases the risk of clogging. Wax (paraffin) is a heavy organic compound consisting mainly of high molecular weight paraffinic compounds that are crystalline in nature and range from C20 to C90.^107^ When wax deposition occurs, it reduces the flow area and rate, and increases the pressure drop.^108^ However, the wax deposition process is much slower compared to gas hydrate formation.^109^

Liao et al., applying molecular dynamics simulations, discovered that the influence of wax molecules on CH_4_ hydrate formation is intricate, largely depending on how wax molecules and gas bubbles are distributed within the system. A dual effect of wax on hydrate formation was observed, with a promoting influence during the early stages of the simulation and an inhibitory effect in the middle to later stages. Notably, increasing the number of wax molecules shifted the inhibitory effect to a promotional one. The presence of wax molecules promotes the formation of gas bubbles, as CH_4_ and wax molecules are attracted to each other due to their hydrophobic nature and miscibility. This leads to wax molecules being incorporated into bubbles or CH_4_ aggregating around them. Once gas bubbles form, the rate of hydrate growth decreases. The presence of wax molecules in the gas bubble can affect the distance between the gas–solid interface. A smaller gas–solid interface distance facilitates the stabilization of the hydrate growth interface, as it increases the likelihood of contact between the bubble and the hydrate phase. This interaction enhances the hydrate growth process, with gas–liquid–solid phase mass transfer playing a key role in the overall hydrate formation.^110^

Later, Liao et al. demonstrated that the varying growth pathways of CH_4_ hydrate with wax molecules are likely linked to changes in the mass transfer process of CH_4_ molecules and the structural properties of the interfacial water molecules.^111^ When *n*-heptadecane wax molecules (C_17_H_36_) were placed near the oil–water interface, they inhibited hydrate growth by adsorbing CH_4_ molecules in the oil phase, thereby preventing CH_4_ from migrating to the water phase. In contrast, the addition of methyl heptadecanoate wax molecules (C_18_H_36_O_2_) extended the hydrate growth period, leading to a higher amount of hydrate formation by promoting the conversion of the water film between the hydrate phase and oil phase into hydrate. The cocrystallization of C_17_H_36_ and C_18_H_36_O_2_ inhibited the mass transfer of CH_4_ molecules at the oil–water interface, reducing the hydrate growth rate. This occurred due to a decrease in the available free space after wax absorption and the repulsive interaction between C_18_H_36_O_2_ and CH_4_ molecules.^111^

Li et al. also demonstrated that wax crystals have a dual role in CH_4_ hydrate formation, which depends on their size. Small wax crystals shorten the hydrate nucleation time, promote the formation of the 5^12^6^2^ cages, and increase the conversion rate of CH_4_ and water molecules to CH_4_ hydrate by more than 1.5 times. However, when the size of the wax crystals exceeded a critical threshold, hydrate formation was inhibited, and nucleation time was extended. In this scenario, the competition for CH_4_ adsorption between the wax crystals and the aqueous solution became the dominant factor in the process. The small wax crystals partially facilitated the molecular migration of water and CH_4_ and promoted hydrate formation. However, an increased amount of wax resulted in the aggregation of CH_4_ molecules into nanobubbles, which significantly lowered the CH_4_ concentration in the aqueous solution, reducing it well below the threshold required for hydrate formation.^112^

## 4 Minimizing Risk

To address flow assurance challenges caused by gas hydrate formation in pipelines, the oil and gas industry employs various physical and chemical methods. Physical techniques involve applying thermal heating or depressurization to gas hydrates. Alternatively, chemical inhibitors such as thermodynamic hydrate inhibitors (THI), kinetic hydrate inhibitors (KHI), and anti-agglomerates (AAs) are injected into pipelines to influence the nucleation, growth, or agglomeration of gas hydrates.

### 4.1 Physical Strategies

A variety of physical methods have been proposed to prevent and manage gas hydrate formation in pipelines, including thermal stimulation (heating), depressurization, dehydration, or a combination of these techniques. Since the stability of gas hydrates is closely tied to the temperature and pressure conditions within reservoirs, production strategies are heavily guided by the corresponding pressure–temperature (*p*–*T*) diagrams of those environments, as shown in Figure 4 for CH_4_ hydrate. The method involving temperature elevation is termed “thermal stimulation”, while the technique involving pressure reduction is referred to as the “depressurization” method. “Dehydration” stands out as a highly effective permanent solution for inhibiting gas hydrate formation, as proposed in various studies. The concept is simple: by removing all water from the gas stream, gas hydrates cannot form. However, this technique is deemed unfeasible due to the considerable challenge of completely eliminating water from the gas stream. Additionally, there is a novel technology involving antihydrate surfaces, where advanced surface modification techniques and innovative surface designs offer alternative strategies to address the issues associated with gas hydrate formation. Detailed information about each of the aforementioned methods is provided below. A summary of the literature review on physical strategies to minimize the risk of gas hydrate formation with respect to flow assurance is also given in Table 1.

**Figure 4:**
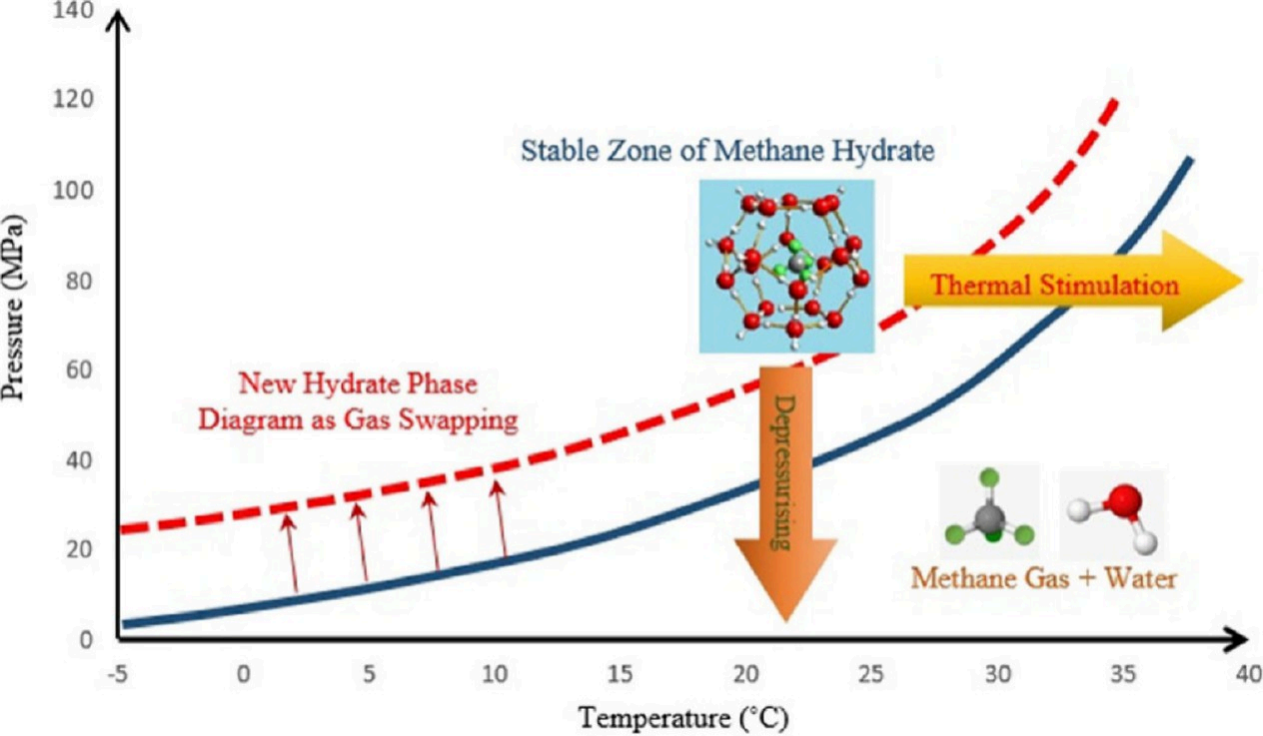
Schematic CH_4_ hydrate phase diagram. This figure was reproduced with permission from ref 113. Copyright 2017 Elsevier.

**Table 1:** Summary of Literature Reviews on Physical Strategies to Minimize the Risk of Gas Hydrate Formation with Respect to Flow Assurance

method	initial system	system size (nm)	simulation time (ns)	short description	reference
depressurization	sI CH_4_ hydrate–vacuum	2.375 × 2.375 × 4.751	0.5	CH_4_ hydrate dissociation was studied at 277 K by the “vacuum removal method” and the normal method	Yan et al.^116^
depressurization	sI CH_4_ hydrate–vacuum	3.64 × 3.64 × 10	300	at 271 K, the simulation below the ice point was carried out, resulting in an initial pressure of 2.5 MPa and a subsequent drop to 1.3 MPa	Naeiji et al.^33^
heating	sI CH_4_ and CO_2_ hydrate	∼2.4 × 2.4 × 24	0.6–1.1	the temperature was raised to 370 K with rates of 0.1–20 TK/s after the simulation began at 270 K and 5.0 MPa	Iwai et al.^118^
heating	sI CH_4_ hydrate	∼4.8 × 4.8 × 4.8	5	under various temperature ramping scenarios, an initial temperature of 100 K and a final temperature of 500 K were employed, alongside a constant pressure of 5 MPa	Duenas^119^
heating	sI CH_4_ hydrate–vacuum	3.64 × 3.64 × 10	200	the system temperature gradually increased from 274 K and the pressure was 7 MPa until the hydrate phase dissociation was completed (318 K)	Naeiji et al.^33^
heating	sII binary (CH_4_ + C_3_H_8_) hydrates and sII mixed (CH_4_ + C_2_H_6_ + C_3_H_8_ + CO_2_) hydrates–vacuum	3.54 × 3.5 × 10	225	the system’s temperature progressively ascended from 278 to 323 K, under a pressure of 3 MPs, until the hydrate phase had completely dissociated	Pan et al.^127^
heating	sI CH_4_ hydrate–water phase	∼3.6 × 3.6	0.4	at initial simulation temperatures of 273, 290, 300, and 310 K, dissociation took place under 10 MPa of pressure	Bagherzadeh et al.^120^
heating	sII C_3_H_8_ and binary (CH_4_ + C_3_H_8_) hydrates–water phase	13.848 × 3.462 × 3.462	50	various starting temperatures of 292, 302, 312, and 322 K were carried out at 3 MPa of equilibrium pressure	Yang et al.^123^
heating	sI CH_4_ hydrate–water–with/without nanobubble	43.08 × 21.54 × 3.61	200	the effect of temperature (292, 302, 312, and 322 K) with/without nanobubbles was evaluated, while the pressure was maintained at 0.1 MPa	Fang et al.^124^
heating	sI CH_4_ hydrate–water/CH_4_ aqueous phase	∼6 × 6 × 12	∼30	the melting curve is computed for the SPC/E, TIP4P/2005, and TIP4P/Ice Water models using direct coexistence simulations over a broad range of pressures up to 5000 bar	Smirnov and Stegailov^125^
heating	sI CO_2_ hydrate–water phase	∼9.6 × 9.6 × 5	8	the thermal-driven breakup of the planar hydrate interface in liquid water at 300–320 K has been studied using equilibrium and non-equilibrium MD simulations	English and Clarke^54^
heating	sII binary (CH_4_ + C_3_H_8_, CH_4_ + i-C_4_H_10_, CH_4_ + C_3_H_8_ + i-C_4_H_10_) hydrates	3.56 × 3.56 × 3.56	0.7	the effects of temperature (280–340 K), pressure (20–70 MPa), cage occupancy, and inhibitor (methanol, ethanol, glycerol) on the decomposition phenomenon were analyzed	Kondori et al.^126^
antigas hydrate surface	sII CH_4_ and THF hydrate–THF solution–CH_4_ + Ni foam + hydrophobic silica + hydrophobic multihydroxyl polymer [P(HHIP)]	5.9 × 8.1 × 8.5	500	a strong 3D porous skeleton that is superhydrophobic and resistant to hydrate nucleation was simulated at 250 K and 500 bar	Yin et al.^131^
antigas hydrate surface	sI CH_4_ hydrate–*n*-alkane or alcohol molecules (C_*n*_ or C_*n*–1_OH)–amorphous water/CH_4_	5.16 × 5.16	200	antihydrate surfaces were simulated at 210 K by creating a gas coating to reduce hydrate adhesion	Ma^133,134^
antigas hydrate surface	sI CH_4_ hydrate–water/CH_4_–solid surface	∼3.6 × 12	200	it was simulated with different gas concentrations in the aqueous phase, surface roughness, and temperatures (210, 220, 230, and 250 K)	Ma^135^

#### 4.1.1 Depressurization

Depressurization is a remedial technique that involves lowering the pressure on one end of a gas pipeline to create a pressure gradient, encouraging hydrates to migrate toward the lower-pressure side. However, this method is typically used after hydrate formation and does not prevent its occurrence. In high-pressure gas pipelines, depressurization is often impractical and may even accelerate the movement of hydrate plugs, posing a risk of damaging the pipeline. Additionally, as gas is released and hydrates dissociate, the resulting drop in temperature can impact both gas flow and the progression of hydrate dissociation.^114,115^

Due to the inherent challenges associated with inducing hydrate dissociation through pressure reduction, there are limited reports available on the decomposition mechanisms using MD simulation. Yan et al. conducted an investigation into CH_4_ hydrate dissociation via depressurization using MD simulation. It was found that hydrate dissociation is promoted by depressurization. The driving force for hydrate dissociation is governed by the concentration gradient between water molecules in the surface hydrate layer and those in the inner layers. This leads to a gradual breakdown of the clathrate structure, causing the hydrate to decompose progressively, layer by layer. The study revealed that dissociation proceeds more slowly under pressure reduction compared to thermal stimulation or the use of chemical inhibitors.^116^ Naeiji et al. observed that depressurization below the ice melting point led to water molecule recrystallization into either hydrate-like structures or ice. This process created a barrier that inhibited further dissociation, resulting in a self-preservation phenomenon in structure I CH_4_ hydrate. The inner layers of the hydrate remained largely encapsulated by either ice or a sluggish amorphous water phase, effectively stopping continued decomposition. New crystal structures formed below the ice point, accompanied by the formation of a quasi-liquid or amorphous water layer as the outer hydrate structure disintegrated. Unstable partial hydrate cavities temporarily slowed or halted gas release.^33^

Another technique that offers a significant new technology for hydrate plug remediation is the nitrogen purge plug dissociation method, in which the hydrate is gas-permeable. Even though the temperature and pressure do not change, the hydrate former’s decreased chemical potential in the gas phase encourages hydrate dissociation. When depressurization is not an option or is difficult to implement, this approach is practical and can be utilized.^117^

#### 4.1.2 Heating

Thermal stimulation involves raising the temperature above the equilibrium temperature at given pressure. In this process, it is essential to ensure that the energy input required for hydrate decomposition and gas production remains lower than the energy that can be extracted from the released gases. Only then can the operation be considered economically viable.^21^

In MD studies of hydrate dissociation, a range of geometries have been explored. However, the influence of various factors on the hydrate decomposition process remains poorly understood. These include the specific composition of the hydrate and its cages, the effects of temperature elevation or other external perturbations, guest molecule transfer from open cavities into the liquid phase, and the mechanisms of thermal dissipation at the gas–hydrate or liquid–hydrate interface. Each of these elements may significantly impact the rate and stability of hydrate breakdown. Iwai et al. investigated the dissociation processes of CH_4_ and CO_2_ hydrates by heating the system up to 370 K, with temperature ramp rates ranging from 0.1 to 20 TK/s. Their findings indicated that the water cages collapsed initially, after which gas molecules began to escape. The dissociation temperature depended on the rate of temperature increase, with CO_2_ hydrate dissociating at lower temperatures and faster rates compared to CH_4_ hydrate. CH_4_ hydrate demonstrated greater stability under the tested conditions.^118^ Duenas also investigated the dissociation conditions of CH_4_ hydrate by varying heating rates from 0.8 to 400 TK/s and temperature increments above equilibrium conditions. Results showed dissociation temperature increased with higher heating rates. Dissociation appeared uniform across the structure with no clear preference between large and small cages. At a high heating rate of 400 TK/s, oxygen atoms in water molecules exhibited behavior similar to CH_4_ gas, possibly indicating rapid evaporation. Slower heating rates showed CH_4_ molecules behaving more diffusively.^119^ Some studies have also indicated a preference for cavity breakdown during gas hydrate dissociation, as demonstrated in the study by Naeiji et al.^33^ As long as significant portions of the hydrate phase remained intact, the proportion of CH_4_ molecules in the large and small cages remained stable. As the hydrate phase underwent considerable decomposition, the remaining hydrate structure likely fragmented into clusters of hydrate cages. This fragmentation resulted in a tendency for the tetrakaidecahedra (5^12^6^2^) cages to break up more readily than the pentagonal dodecahedra (5^12^) cages.^33^

Bagherzadeh et al. carried out constant-energy molecular dynamics simulations to investigate the endothermic decomposition of CH_4_ hydrate in the presence of water. Their study focused on understanding how mass and heat transfer phenomena influence the rate of hydrate decomposition. They observed that the decomposition proceeds in a series of steps in which the hydrate layers are decomposed one after the other. As the clathrate cages disintegrate, temperature gradients of up to 20 K may form across various regions of the solid and dissolved hydrate phases. These gradients can drive substantial heat transfer between the surrounding aqueous environment and the surface of the hydrate, influencing the overall decomposition dynamics.^120^ Also, the simultaneous release of CH_4_ gas from the decomposing layers of hydrate leads to the formation of CH_4_ nanobubbles in the water phase, which has been confirmed by several studies.^121,122^ Yang et al. reported that, upon release from the hydrate structure, guest gas molecules rapidly formed nanobubbles. In binary hydrate systems, these nanobubbles merged together, ultimately developing into a continuous gas phase as a result of the elevated concentration of gas molecules.^123^ According to Fang et al., hydrate dissociation does not invariably result in the formation of nanobubbles. However, the formation and presence of nanobubbles near the hydrate surface can enhance the hydrate dissociation rate by facilitating directional CH_4_ transfer.^124^ Smirnov and Stegailov also reported the formation of nanobubbles during the melting of CH_4_ hydrate, investigating conditions at pressures up to 5000 bar using various water models. Their study determined the kinetic stability boundary for the sI hydrate, revealing that this structure can withstand substantial superheating. This finding stands in contrast to the predictions of classical nucleation theory, highlighting a greater kinetic resilience of hydrate structures under extreme conditions. The study revealed an universal relationship between the stability boundary, heating rate, and system volume, showing that decreased cage occupancy lowers the decay temperature.^125^

MD simulations conducted by English and Clarke explored the microscopic mechanisms behind the dissociation of CO_2_ hydrates triggered by conventional heating and included a fluctuation–dissipation analysis of the process. Their findings indicated that dissociation rates were highly temperature-dependent, with considerably higher rates observed at increased overtemperatures relative to the melting point. Moreover, the study confirmed the applicability of the Onsager hypothesis during the initial stages of dissociation, as evidenced by statistically meaningful variations in relaxation times associated with oscillation and dissipation behaviors.^54^

The stability and breakdown of sII hydrates formed by CH_4_, C_3_H_8_, and iso-C_4_H_10_ under a range of temperature and pressure conditions was examined by Kondori et al.^126^ They investigated how clathrate hydrates of different hydrocarbons dissociate at varied temperatures due to gas type and composition differences, with constant pressure and dissociation time. Molecular dynamics (MD) simulations revealed that increasing temperature and decreasing pressure destabilize gas hydrate structures. MD results also indicated a decrease in hydrate structure density with rising temperature. At temperatures exceeding the decomposition threshold, alkane molecules exhibit greater mobility compared to water, leading to higher diffusion coefficients.^126^ Yang et al. also carried out molecular-level investigations into the dynamic dissociation mechanisms of sII hydrates, aiming to support gas recovery strategies from marine hydrate reservoirs. They studied pure C_3_H_8_ and binary C_3_H_8_–CH_4_ sII hydrates in a liquid water environment. The results showed that higher initial temperatures accelerated dissociation rates regardless of hydrate structure or guest occupancy. As dissociation is endothermic, system temperature drops, slowing further dissociation.^123^ In the study conducted by Pan et al., it was observed that a quicker breakdown of small hydrate cavities (5^12^) occurred, which led to an overall increase in the ratio of large to small cavities as decomposition progressed.^127^ Furthermore, the results revealed that CH_4_ molecules were released more rapidly than C_3_H_8_ during the dissociation of the sII CH_4_– C_3_H_8_ hydrate. This behavior is likely due to the higher diffusivity of CH_4_ at the hydrate surface and its comparatively weaker ability to stabilize hydrate cavities. Similarly, in sII mixed hydrates, CH_4_ was released faster than CO_2_ and C_2_H_6_, leading to dynamic changes in the hydrate’s composition over the course of the dissociation.^127^

#### 4.1.3 Antigas Hydrate Surface

The prevention and safe removal of hydrate plugs in deep-water flow assurance are identified as key challenges. Recent progress in surface-modification technologies and innovative surface designs presents promising alternative approaches to mitigate the issues associated with gas-hydrate formation. The concept of antihydrate surfaces, which aim to prevent hydrate formation, is explored with a focus on being “hard to form, weak to deposit, easy to remove” for hydrates.^128^ These surfaces are classified into three categories based on their functioning principles: antihydrate nucleation surfaces, antihydrate deposition surfaces, and low hydrate adhesion surfaces.^128,129^

To design antihydrate surfaces that prevent hydrate nucleation, it is crucial to eliminate the promoting effects observed in simulations. This can be accomplished by modifying surface chemistry or enhancing the surface hydrophobicity. Avoiding surface roughness and pore structures, unless they can trap oil to exclude gas molecules, is also important. Other strategies involve modifying the flexibility of coatings, adding non-ice-binding components to the surface, creating barriers to prevent gas absorption, and integrating kinetic hydrate inhibitors into the surface design. These passive methods show promising results for hydrate mitigation and merit further exploration.^128,129^ The interface and surface characteristics of solid substrates, including hydrophilicity, hydrophobicity, surface roughness, adsorption potential from layer accumulation, crystallinity, and surface layer charge, are all recognized as factors influencing the process of hydrate nucleation. These properties influence the behavior of gas and water molecules, thereby affecting the conditions under which hydrate formation occurs.^130^ Yin et al. proposed a solution to avoid hydrate blockage in pipelines: a strong antihydrate-nucleation superhydrophobic 3D porous skeleton.^131^ Traditional superhydrophobic surfaces reduce adhesion to formed hydrates but can promote new hydrate nuclei formation. The 3D porous skeleton increases the terminal hydroxyl (inhibitory groups) content while maintaining superhydrophobicity. This increase in inhibitory groups inhibits the formation of fresh hydrates while retaining antiadhesive properties toward formed hydrates. MD simulations further support this concept by demonstrating that terminal hydroxyls on superhydrophobic surfaces cause water molecules to be rearranged, inhibiting the hydrate formation.^131^

Five main factors influence the hydrate deposition process: (I) the driving force for hydrate formation, (II) the quantity of adhesive water, (III) the surface property (or anti-agglomerant concentration), (IV) the surface mass transfer coefficient, and (V) the flow shear rate.^132^ Figure 5 presents a schematic illustrating the influence of these factors on the deposition process. Hydrate adhesion and solidification occur in two distinct modes: hydrate-induced growth and competition between hydrate and ice. Mechanical testing shows that ice has an adhesion strength roughly five times greater than the weakest hydrate adhesion strength.^132^ Ma investigated the use of the interfacial gas-enrichment technique in the design of antihydrate surfaces.^133^ This entails producing a “gas coating” to lessen hydrate adhesion and facilitate pipeline flow’s automatic detachment. Ma’s research examines important factors such as temperature, gas content, and surface roughness as well as hydrate adhesion to smooth surfaces. Unlike macroscale roughness, nanoscale roughness acts as crack initiators, reducing ice/hydrate adhesion compared to smooth surfaces. Hydrophilic functional groups, like hydroxyl (−OH) groups, enhance adhesion through hydrogen bonding, which changes the adhesion state from adhesive to cohesive failure and increases the adhesion strength.^133,134^ In another work, they investigated how surface engineering could minimize hydrate adhesion, proposing that optimal surface roughness may offer a new approach to developing antihydrate materials. Moreover, a classifier-like relationship between hydrate adhesion strength and nanoscale interfacial structures was discovered. This classifier makes it easier to use machine learning to identify promising antihydrate surface materials on a large scale.^135^

**Figure 5:**
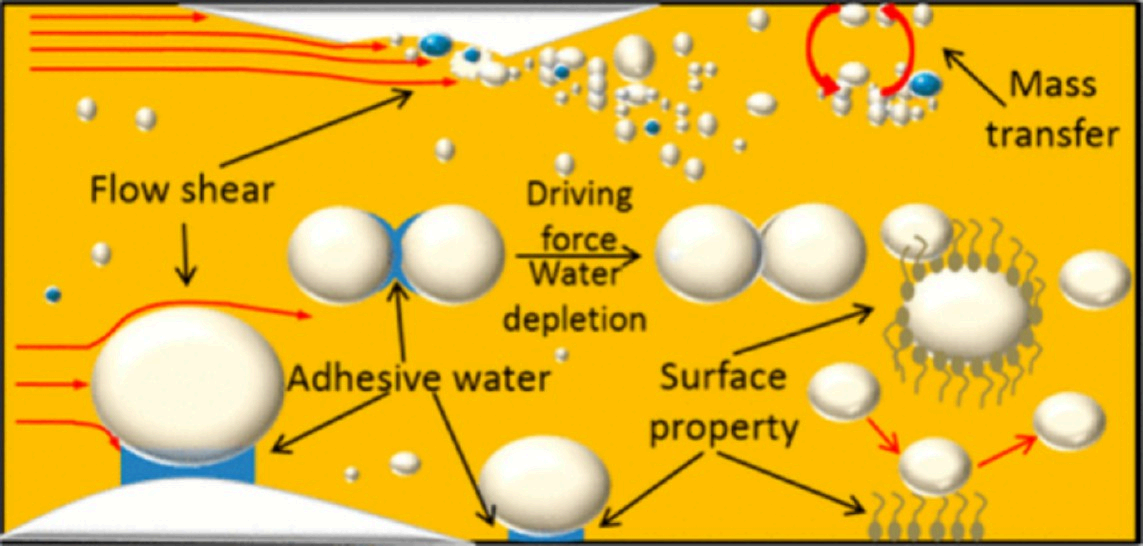
Schematic diagram of the variables that affect the hydrate deposition process. This figure was reproduced with permission from ref 132. Copyright 2017 American Chemical Society. Surface free energy, gas wettability, and surface structure are among the surface characteristics taken into account for antihydrate deposition. The white bubbles, blue surfaces, and gray shapes show the hydrates, water, and additives, respectively.

### 4.2 Chemical Strategies

Chemical strategies for mitigating and inhibiting gas hydrates are considered to be more economical and effective as compared to physical methods which are highly dependent on the local pipeline conditions and usually require high energy consumption and challengeable maintenance.^13^ The most well-known approaches involve the use of chemical inhibitors, which can be injected at specific points in the pipeline, offering a more versatile and cost-effective approach to maintain the pipeline as a hydrate-free region. Generally, they can be classified into two main categories depending on the mechanisms of action: high-dosage thermodynamic hydrate inhibitors (THIs), and low-dosage chemical inhibitors [kinetic hydrate inhibitors (KHIs) and anti-agglomerants (AAs)]. Each category of chemical additives will be discussed in the following sections separately and summarized in Table 2.

**Table 2:** A summary of the literature reviews on chemical strategies to minimize the risk of gas hydrate formation with respect to flow assurance

method	chemical	initial system	system size (nm)	time scale (ns)	short description	reference
THI	methanol	aqueous solution of water and CH_4_ by melting sI CH_4_ hydrate + glycol	∼4.8 × 4.8 × 4.8	2000	methanol disrupts water hydrogen bonding, and can even enter the cages temporarily	Lu et al.^138^
THI	methanol	water/methanol solution + CO_2_ gas phase	4 × 4 × 12	100	methanol preferentially accumulates at the gas–liquid interface, affecting gas solubility and hydrate nucleation	Sujith et al.^144^
THI	ethanol	sI CH_4_ hydrate + water/ethanol solution	3.8 × 3.8× 4.8	12	the addition of ethanol promotes the production of methane bubbles and speeds up the breakdown of CH_4_ hydrates; the hydrate dissociation rate increases with an increasing ethanol concentration	Sun et al.^145^
THI	methanol, ethanol, 1-propanol, 1-butanol, 1-pentanol, 2-pentanol, 3-pentanol	sI CH_4_ hydrate + alcohol layer	2.375 × 2.375 × 4.751	2.1	short-chain alcohol helps to accelerate the breakdown of methane hydrate	Dai et al.^146^
THI	ethanol, 1-propanol, 2-propanol	sII binary CH_4_–alcohol hydrate + alcohol solution	3.462 × 3.462 × 3.462	0.75	alcohols act as both hydrogen bond donors and acceptors, forming transient but long-lived hydrogen bonds with water molecules in the hydrate cages and may act as a hydrate promoter	Alavi et al.^147^
THI	ethanol	sI ethanol–CO_2_ hydrate–ethanol solution	3.627 × 3.627 × 3.627	1.1	ethanol acts as a promoter at low concentrations by lowering the formation pressure, but becomes an inhibitor at higher concentrations due to destabilization of the hydrate structure when adjacent cages are occupied	Alavi et al.^148^
THI	ethylene glycol	sI CH_4_ hydrate + ethylene glycol solution	3.7 × 3.7 × 9.0	0.5	the hydrate structure dissociates as a results of the hydroxyl groups in ethylene glycol breaking the hydrogen bonding network inside it	Hembram et al.^150^
THI	NaCl	aqueous solution of Na^+^ and Cl^–^ and CH_4_	6 × 6 × 6	2000	when ions restrict the surrounding water molecules, a hydration shell forms; ions also increase the apparent concentration of CH_4_ but inhibit their diffusion	Bai et al.^152^
THI	NaCl, KCl, CaCl_2_	salt aqueous solution with CH_4_ + porous medium	4 × 3.6 × 9.7	3000	salt prevents methane hydrate formation by reducing the amount of free water molecules available for hydrate cage formation	Wu et al.^151^
					the inhibition effectiveness: KCl > CaCl_2_ > NaCl	
THI	NaCl, KCl, CaCl_2_	sI CH_4_ hydrate + salty solution	∼2.4 × 2.4 × 3.6	0.6	more inorganic ions could shorten the stagnation time; the ionic capacity to break down hydrate cells is displayed in an orderly sequence, demonstrating the superiority of calcium ions (Ca^2+^) over potassium ions (2K^+^) and chloride ions (2Cl^–^), followed by sodium ions (2Na^+^)	Xu et al.^153^
THI	NaCl	sI CH_4_ hydrate + salty solution	larger than 10.8 × 10.8 × 10.8	120	NaCl has two opposing effects on methane hydrate dissociation: initially slows dissociation by stabilizing the hydrate interface but later accelerates dissociation due to rapid methane bubble formation	Yagasaki et al.^154^
KHI	PVP, PVCap	sI CH_4_ hydrate + propane/PVCap solution	2.4 × 2.4 × 10.6	0.9	PVCap has stronger hydrate interaction than PVP	Kvamme et al.^161^
KHI	PVCap	sI ethylene oxide hydrate + ethylene oxide solution	9.3 × 9.8 × 13.1	500	the effectiveness of PVCap in inhibiting hydrate growth is attributed to the Gibbs–Thomson effect, where an increase in surface curvature leads to a decrease in the crystal growth rate	Yagasaki et al.^163^
KHI	PVP, PVP–A, PVP–ME, PVP–EE, PVP–PE, PVP–BN	sI CH_4_ hydrate + CH_4_/water solution	2.3786 × 2.3786 × 10.8	140	hydrophobic butyl and ester group on PVP molecule enhances inhibition efficiency	Cheng et al.^164^
KHI	asparagine, serine, PVP, PVCap, PNIPAM, pDMAEMA, PHEMA, PEG	CH_4_/KHI solution	3.94 × 3.60 × 11.70	4	asparagine was the most effective kinetic inhibitor, suppressing methane hydrate formation by dissolving more in bulk water rather than accumulating at the methane–water interface	Oluwunmi et al.^160^
KHI	C_60_	CH_4_/C_60_ solution	4.3 × 4.3 × 4.3	200	C_60_ molecules significantly prolong the induction time, reduce hydrate formation rate, and lower the total amount of hydrate produced	Liu et al.^168^
KHI	ionic *N*-vinyl caprolactam/maleic-based copolymers	sI CH_4_ hydrate + CH_4_/KHI solution	∼2.4 × 2.4 × 2.4	100	the inhibitors effectively adsorb onto both hydrate and metal surfaces, thereby impeding gas hydrate formation and mitigating corrosion	Omidvar et al.^165^
KHI	DPI2	sII CH_4_ + C_2_H_6_ + C_3_H_8_ + i-C_4_H_10_ + *n*-C_4_H_10_ + CO_2_ + N_2_ hydrate + aqueous phase with KHI	5.41 × 5.33 × 9.09	20	the transportation of gas molecules to the growing hydrate cages was partially covered by DPI2 adsorption on the hydrates’ surface, which served as a barrier to mass transfer and caused disruption	Farhadian et al.^166^
KHI	AAI	sI CH_4_ hydrate–water/methanol solution	5.4 × 5.4 × 0.8	2000	the hydrate adhesion is significantly weaker in the presence of AAI, demonstrating its dual functionality as both a gas hydrate inhibitor and a corrosion inhibitor	Hu et al.^167^
KHI/THI	EMIM-Cl	sI CH_4_ hydrate + EMIM-Cl solution + gas phase	∼2.4 × 2.4	41.2	EMIM-Cl forms hydrogen bonds with water molecules, disrupting the orderly arrangement necessary for hydrate formation, while its bulky structure physically impedes the proper alignment of water molecules, further hindering the growth of hydrate cages	Xin et al.^175^
KHI/THI	benzene, EMIM-Cl, methanol, NaCl, THF	CH_4_ + water + additive in CNT	CNT of 5.0	26	EMIM-Cl is the best inhibitor from both kinetic and thermodynamic aspects; the structural properties of CNTs, such as chirality and flexibility, significantly influence the behavior and effectiveness of hydrate inhibitors	Abbaspour et al.^174^
KHI/THI	C_2_OHmim]f_2_N, C_3_(OH)_2_mimf_2_N, C_2_mimBF_4_, C_4_mimBF_4_, C_4_mimOA_C_, C_4_mimE_t_SO_4_	sI CH_4_ hydrates + CH_4_/additive solution	5.0 × 2.4 × 2.4	35	these ILs are found to act as dual function hydrate inhibitors; the effect of cation type on the kinetic inhibition effectiveness is more significant than that of anion type	Haji Nasrollahebrahim et al.^176^
KHI/THI	[N_2 2 2 2_]Br, [N_4 4 4 4_]Br	sI CO_2_ hydrates + additive solution	4.7 × 4.7 × 4.7	2	the anion–cation interaction in [N_2 2 2 2_]Br is stronger; the distinct function of ILs may be primarily due to the Br^–^ anion’s decreased propensity to cross-link with water molecules to form semiclathrate hydrates	Wang et al.^178^
KHI	alanine, proline, glycine, serine	sI CH_4_ hydrates + CH_4_/ additive solution	2.4 × 2.4 × 7.2	30	the ranking of the inhibitory effect is serine > glycine > alanine ≈ proline; serine and glycine are more effective due to their unique chemical structures, high solubilities, strong hydrogen bond capabilities and low hydrophobicity	Maddah et al.^182^
KHI	serine, glycine, valine	sI CH_4_ hydrates + additive	2.324 × 2.324 × 3.486	0.8	the interaction of amino acids with hydrate is governed by both electrostatic interactions arising from the side chains and the ability to form hydrogen bonds; of the three amino acids studied, serine shows the best inhibitory impact	Hu et al.^183^
KHI	Ala-Ala, Ala-Gly, Gly-Gly	sI CH_4_ hydrate + additive solution	3.6 × 2.4 × 8.0/4.8 × 5.9 × 12	350/40	the N-termini of each dipeptide are the core components that had the most decisive impact on the hydrate slab; Ala-Gly interact most strongly with the CH_4_ hydrate	Go et al.^184^
KHI	alanine-rich short peptides	sI CH_4_ hydrate/CH_4_ solution/gas phase	5 × 5 × 5	200	the presence of dual methyl groups in alanine facilitates effective docking onto the hydrate surface, thereby inhibiting hydrate growth	Li et al.^185^
KHI	alanine-rich peptides (5AD, 5AK, 5AT, 5AA)	sI CH_4_ hydrate + gas/additive solution	3.1043 × 3.1043 × 3.1043	400	incorporating threonine into the peptide structure further enhanced the inhibitory effect	Chen et al.^186^
KHI	AFP III and other mutants (N14S, T18N, Q44T, AAA)	sI CH_4_ hydrate + CH_4_/additive solution	7.2 × 4.8 × 9.8	30	the antifreeze activity of AFP III is influenced more by the length and shape of the side chains of certain amino acids rather than their hydrogen-bonding capabilities	Maddah et al.^192^
KHI	AFP I	sI CH_4_ hydrate + CH_4_/additive solution	8.4 × 3.6 × 9.8	30	when type I AFP adsorbs, the hydrate surface bends around residues 15–17, creating a curvature at the hydrate/water interface; this deformation contributes to its adsorption–inhibition mechanism and hinders mass transfer	Maddah et al.^193^
AA	quaternary ammonium salt (QAS) and QA cation (QAC)	sII CH_4_–C_3_H_8_ hydrate–water/gas solution	4.9 × 4.3 × 16.3	150	the results highlighted the difference between the nature of anti-agglomerant/hydrate interactions as compared to kinetic inhibitor/hydrate interactions	Bellucci et al.^197^
AA	*n*-dodecyl-tri(*n*-butyl)-ammonium chloride	sII CH_4_–C_3_H_8_ hydrate + gas/additive solution	4.8 × 4.2 × 8	7200	in higher salinity environments, the surface adsorption of the AAs is enhanced	Mehrabian et al.^198^
AA	AAC8, AAC12, AAC121, AAC171	sII CH_4_/C_2_H_6_ hydrate + AA solution + gaseous and liquid hydrocarbons	11.193× 3.462 × 16	≥200	the simulation system consisted of two hydrate nanoparticles immersed in liquid hydrocarbons (*n*-dodecane or *n*-heptane), with various AAs introduced to evaluate their impact on hydrate cohesion and agglomeration prevention	Phan et al.^199^
AA	AA1–AA4	sII CH_4_–C_3_H_8_ hydrate + AA + water droplet	8.6 × 8.6 × 19	120–600	both steered and non-steered MD are used to investigate the agglomeration behavior of sII hydrates and assess the influence of the anti-agglomerants on this process	Mohr et al.^200^
AA	AA-01 to AA-10	sII CH_4_–C_3_H_8_ hydrate + water layer + fluid with AA solution	5.193 × 5.193 × 17	120	the study found that the thickness of the liquid water layer on the hydrate surface significantly affects the adsorption efficiency of AAs, with thicker layers reducing AA adsorption and an optimal water layer thickness exists for hydrate growth promotion	Mohr et al.^201^
AA	AA contains two long hydrophobic tails, R1 (one *n*-dodecyl chain), and one short hydrophobic tail, R2 (linear hydrocarbon chains of four carbon atoms)	sII CH_4_ hydrate + CH_4_/AA solution	5.193 × 5.193 × 9.962	100–200	the molecular structure of aromatic compounds plays a key role in their interaction with AAs, leading to either synergistic or antagonistic effects on gas hydrate agglomeration; monocyclic aromatics diminish AA’s effectiveness while polycyclic aromatics could enhance the performance of AAs	Bui et al.^203^
AA	1-phenylacetic acid, 2-naphthylacetic acid, 1- pyreneacetic acid	sII CH_4_–C_3_H_8_ hydrate + quasi-liquid layer with AA + hydrocarbon phase	4.9 × 2.15 × 12.5	200	this study shows that polynuclear aromatic carboxylic acid surfactants reduce hydrate particle aggregation by adsorbing onto the hydrate surface and disrupting capillary liquid bridges, with 1-pyreneacetic acid demonstrating the strongest anti-agglomeration effect	Fang et al.^205^
AA	phenylacetic acid, 2-naphthylacetic acid, 1-pyreneacetic acid	sI CH_4_ hydrate + CH_4_/AA solution	4.65 × 4.65 × 15.86	90–180	this study reveals that aromatic carboxylic acids strongly adsorb to hydrate surfaces, especially in hydrocarbon phases; this is primarily due to van der Waals forces between the acid’s aromatic rings and the hydrate surface, indicating their potential as effective AAs	He et al.^206^
AA	BAA1, BAA2	sII CH_4_–C_3_H_8_ hydrate + gas/AA solution	5.14 × 5.14 × 15.4	>10	the headgroup of BAA1 molecules adsorbed onto the hydrate surface, while their alkyl chains extended into the hydrocarbon phase, effectively dispersing hydrate particles and preventing agglomeration	Tang et al.^212^
AA	oleic acid derivatives (OAD)	sI CH_4_ hydrate–water/OAD solution/iron layer	13.4268 × 3.3567 × 10	100	OAD has the potential to serve as an eco-friendly resource for creating dual-function inhibitors that address both hydrate formation and corrosion, contributing to safer hydrate management practices	Tang et al.^214^

#### 4.2.1 Thermodynamic Hydrate Inhibitor (THI)

Thermodynamic hydrate inhibitors (THIs) function by altering the phase equilibrium of gas hydrates, changing their stability conditions toward higher pressure and lower temperatures. This shift makes hydrate formation less likely under typical pipeline environments.^13^ At a molecular scale, they disrupt the highly cooperative hydrogen bonding network of water molecules which are responsible for forming cage-like structures of gas hydrates.^136^ With this regard, THIs such as small alcohols (methanol and ethanol), glycols (ethylene glycol and monoethylene glycol), and salts (NaCl, KCl, and CaCl_2_), are widely introduced into the flow stream.^15,137–139^

Alcohols such as methanol and ethanol are among the most commercially used THIs for their high efficiency, as their molecular structures combines a polar hydroxyl group with a nonpolar alkyl segment. The hydroxyl group forms stronger, directional hydrogen bonds with water molecules, thereby competing with and disrupting the water–water hydrogen bonds in hydrate structures.^140^ This competition decreases the local availability of water molecules in the proper orientation to assemble into the open, tetrahedrally coordinated network needed for hydrate formation. Simultaneously, the hydrophobic alkyl part of the alcohol molecule organizes the surrounding water molecules, leading to the formation of a hydration shell in which water molecules adopt a more “structured” arrangement to minimize contact with the hydrophobic surface.^141^ This reorganization, driven by the need to reduce energetically unfavored interactions, further disturbs the formation of stable hydrogen-bonded network for gas hydrates. By combining these two effects, the disruption of water–water hydrogen bonds by the hydroxyl group and the restructuring of water due to the hydrophobic alkyl chain, the overall water network becomes less favorable for hydrate cage assembly, effectively inhibiting hydrate formation.^18,141^ The behavior of methanol during hydrate nucleation and growth have been illustrated from the MD simulation work by Lu et al. (Figure 6). The methanol molecules shown as blue spheres and sticks were gradually absorbed by the formed cavity surfaces due to their existing hydrophobic groups similar to guest molecules. Despite the system containing a high concentration of guest molecules, the methanol molecules were still able to interact with water clusters by forming hydrogen bonds via their hydroxyl hydrogen and could even form cavities temporarily. Noteworthy, methanol molecules were also found to enter the metastable cavities as guests for a while, as indicated in Figure 7. However, these cavities were not stable since the coordination structures are different. As a result, methanol molecules entered the solution again resulting in the successive breakup of cavities (Figure 6f).^138^

**Figure 6:**
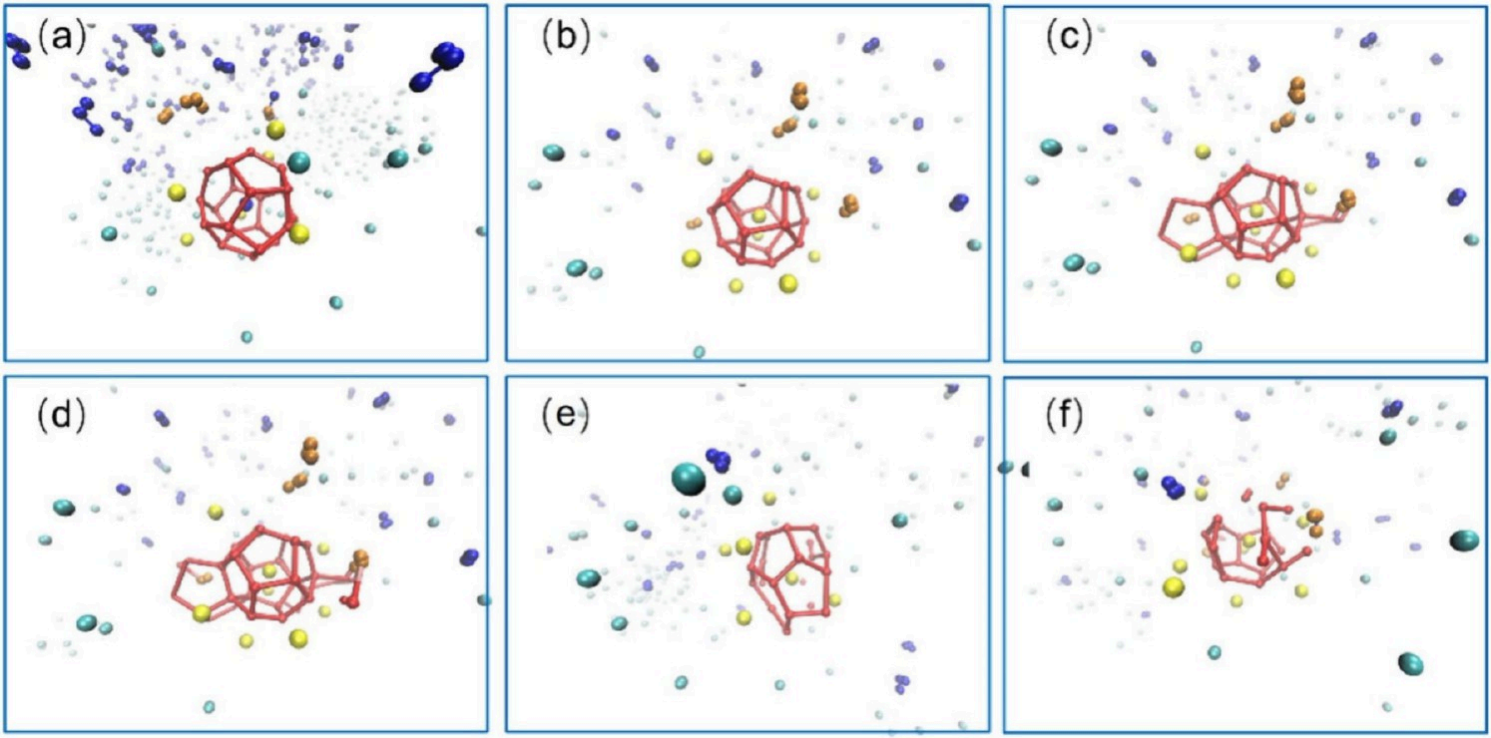
Evolution of unstable cage cluster affected by methanol molecules. (a and b) Single cage forms and methanol molecules approach the cage cluster; (c and d) cage cluster grow; and (e and f) methanol molecules leave the cluster, alter the orientation of surrounding water molecules, and trigger the disintegration of the cage structure. The yellow and cyan spheres represent CH_4_ in the bubble and CH_4_ in the water, respectively. The blue and orange spheres and sticks both indicate methanol molecules while the red ones represent water cages. This figure was reproduced with permission from ref 138. Copyright 2022 Elsevier.

**Figure 7:**
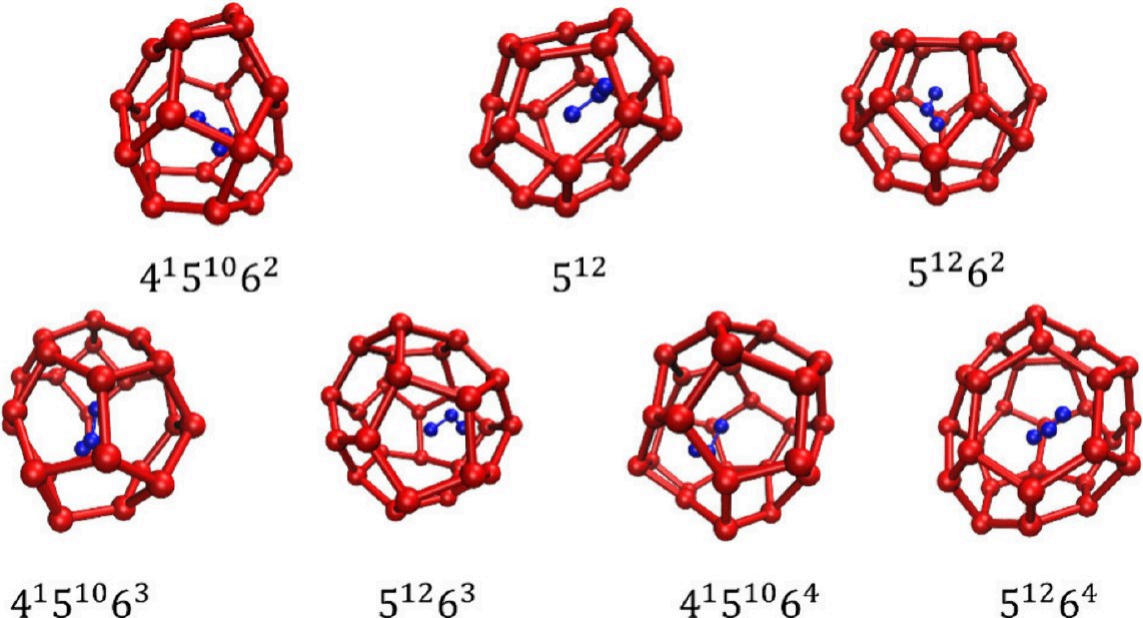
Seven types of cages occupied by methanol. This figure was reproduced with permission from ref 138. Copyright 2022 Elsevier. The blue and red spheres and sticks represent methanol in the cage and water cages, respectively.

Similar visualization evidence has been provided by other researchers,^142,143^ who observed methanol molecules remaining at the edges of the CH_4_ hydrate region with some of them being trapped inside hydrate cavities. A decrease of 1.35% in the number of hydrogen bonds per water molecule have been reported with respect to the pure water system. As an amphiphile, the presence of methanol, or more specifically the hydrophobic methyl group, promotes a more ordered arrangement of water molecules around dissolved gas and makes the local structure more hydrate-like by orienting the methyl group toward the gas molecule. However, as a result of hydrogen bonding between their hydroxyl group and the water molecules they also impede the availability of water molecules around the gas molecules for the formation of gas hydrates.^144^

Ethanol also affects hydrate formation both by disrupting the hydrogen bonding network and by altering water structuring near hydrate-forming regions. Sun et al. demonstrated through MD simulations that increasing ethanol concentrations promote methane hydrate decomposition by accelerating methane gas bubble formation and weakening the water framework. This was attributed to ethanol’s ability to reconstruct hydrogen bond networks and its favorable mass transfer properties, especially at concentrations up to 40 mol %. The study further revealed that increasing temperatures and decreasing pressures enhanced this decomposition effect, indicating ethanol’s strong thermodynamic influence under operational conditions.^145^ Dai et al. explored how the molecular structure of alcohols impact methane hydrate dissociation and found that the amphiphilic character and molecular size of ethanol significantly impacted its inhibitory strength. Specifically, shorter carbon chains and a higher number of the hydroxyl groups enhance the alcohol’s ability to promote methane hydrate decomposition.^146^ In an earlier study, Alavi et al. investigated the role of ethanol, 1-propanol, and 2-propanol in sII methane hydrates and found that their hydroxyl groups function as both proton donors and acceptors, forming simultaneous hydrogen bonds with different cage water molecules. The presence of hydrophobic alkyl groups and the nonpolar methane guest molecules contribute to the stabilization of the hydrate phase. The study suggests that ethanol may also act as a weak hydrate promoter rather than acting as a typical inhibitor, as it perturbs the water network less and maintains overall hydrate stability, especially in the presence of methane and the sII hydrate structure.^147^ A subsequent study by Alavi et al. expanded on this by examining ethanol–CO_2_ binary sI hydrate system, revealing ethanol’s dual behavior. At higher concentrations, ethanol reduces both the occupancy and stability of hydrate cavities due to strong hydrogen bonding with water molecules while disrupting the native water–water hydrogen bonding network, especially when ethanol molecules occupy adjacent large cavities which induces local structure collapse. Interestingly, at lower concentrations, the ethanol molecule can also act as a hydrate promoter by lowering the CO_2_ gas pressure threshold for initiating hydrate formation in comparison to pure CO_2_ hydrates. This may be linked to the enhanced gas solubility and interface structuring.^148^ Collectively, these studies suggest that ethanol’s amphiphilic nature allows it to modulate both the energetic and structural parameters of hydrate formation, making it an effective THI, albeit less potent compared to methanol.

Ethylene glycol is another conventional thermodynamic hydrate inhibitor that has been commercially used in past years. Compared to methanol, it offers the advantage of being effectively recoverable, regenerable, and recyclable.^32^ However, its inhibition of CH_4_ hydrate formation was not as efficient as compared to the system containing methanol due to the differences in their molecular interactions with water. Methanol molecules exhibit a much stronger attraction to water molecules, acting either as a donor or acceptor in hydrogen bonding, and thus more effectively disrupting the water–water hydrogen-bond network necessary for gas hydrate cage formation.^149^ In contrast, while ethylene glycol molecule contains two hydroxyl groups capable of forming hydrogen bonds, its larger molecular size and the more extended structure of its hydrogen-bonding network result in a less pronounced disruption of the water lattice.^32,138^ Similar to the observation with methanol, some ethylene glycol molecule trapped within the cavities of the gas hydrates, others predominantly locate at the interface between the gas and the hydrate phase.^32^ During hydrate dissociation, the hydroxyl group (−OH) of ethylene glycol forms new hydrogen bonds with water molecules, disrupting the hydrate’s existing structure network. This disruption contributes to the destabilization of the gas hydrate structure and eventual dissociation of the gas hydrate. With an increase in ethylene glycol concentration, there is a surge in the availability of hydroxyl groups, this in turn, causes the hydrate structures to dissociate more quickly.^150^

Inhibition of gas hydrates can also be achieved when electrolytes salts are present in liquid water. Most of the MD simulation studies focus on the hydrate formation and dissociation in NaCl solutions as well as other salt solutions like CaCl_2_ or KCl.^151–155^ After hydrate dissociation, a liquid film is often generated on the hydrate surface, which creates extra mass transfer resistance in further decomposition, and leads to the stagnation of the process. Electrolytes (CaCl_2_, NaCl, and KCl) could disrupt the liquid film structure and shorten the stagnant time, as water molecules are more strongly attracted by electrostatic forces to the cations and anions than to the hydrate structure. The water molecules form a hydration shell around the dissolved salt ions and are therefore no longer available for hydrate formation. Bai et al. reported that the presence of NaCl prolongs nucleation induction time and suggested that the electrolyte’s charge (Coulomb interactions) may significantly influence hydrate nucleation.^152^ Water molecules were supposed to move freely and adjust their positions to form cage-like precursors during hydrate nucleation.^156^ Electrolyte ions resisted the movement of water molecules, since a hydration shell could be formed around these ions. Moreover, specific ions play distinct roles: the negatively charged Cl^–^ can interact with water molecules across a broader range of orientation angles as compared to Na^+^, slowing down their exchange with free molecules during hydrate nucleation.^152^ Additionally, the salt promoted the hydrate dissociation especially at high temperatures when salt ions strengthened the capability to break up the local hydrogen-bond network around the hydrate surface.^151^ The rate of hydrate dissociation accelerated as the concentrations of KCl and CaCl_2_ increased, though this trend was not markedly observed with NaCl. The effectiveness in hydrate inhibition was ranked as follows: 20% KCl > 20% CaCl_2_ > 20% NaCl. Similarly, the capability of the ions to facilitate decomposition was ordered as Ca^2+^ > 2K^+^ > 2Cl^–^ > 2Na^+^ (Figure 8).^153^ It should be noted that, even though salts offer a stronger inhibitory effect than methanol and other alcohols, their highly corrosiveness is an obvious drawback.^136^ The minimum concentration of typical THIs required to mitigate the risk of hydrate formation can range from 20–50 wt % of the water mass, making the pipeline operation both expensive and challenging.^136^ Therefore, implementing low dosage hydrate inhibitors (LDHIs), effective at lower concentrations (0.5–3 wt %), has garnered more interests in recent years due to their potential ecological and financial advantages.^157^

**Figure 8:**
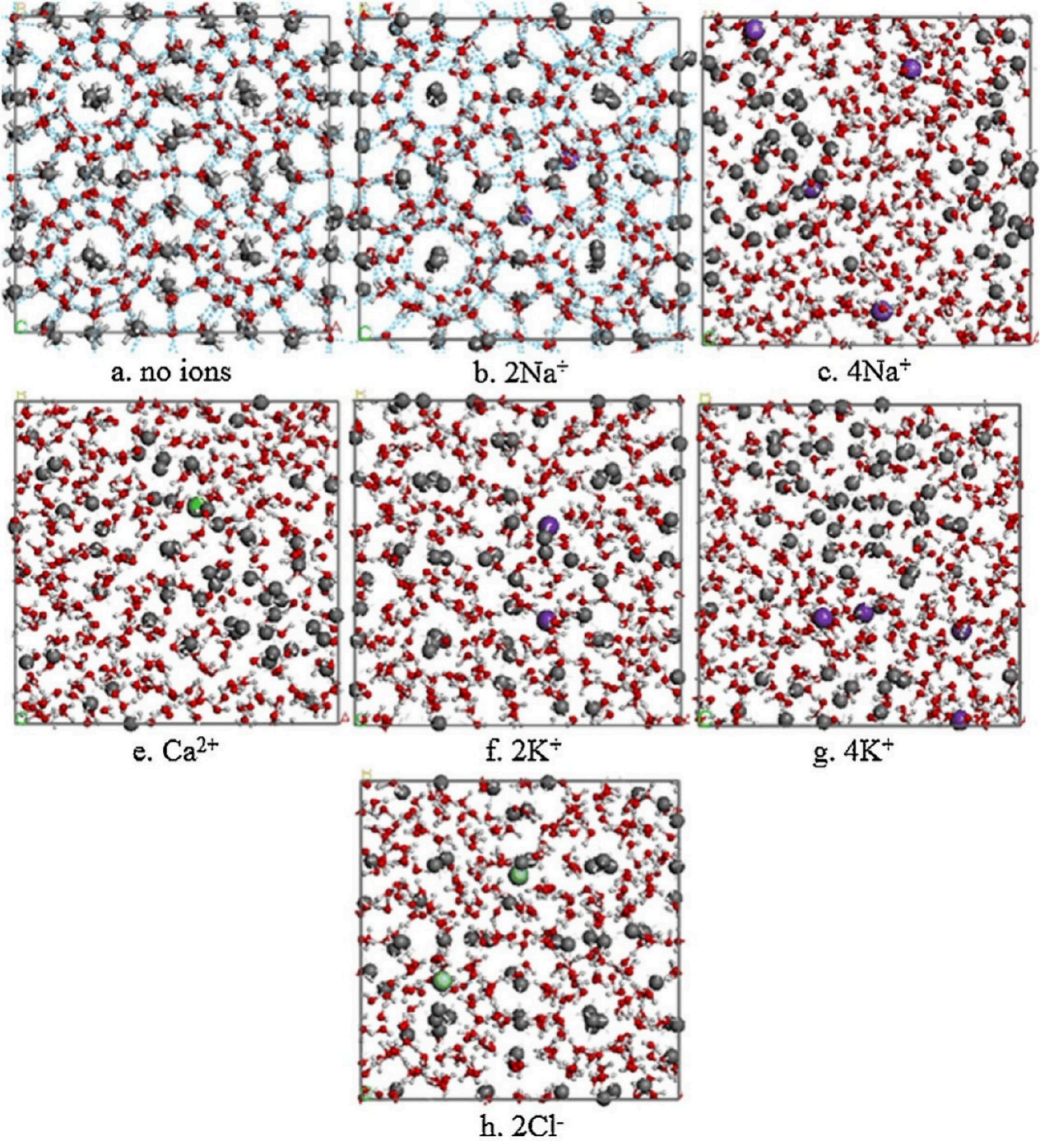
Final configurations of hydrate cells (a) without and (b–h) with inorganic salts. This figure was reproduced with permission from ref 153. Copyright 2017 Elsevier.

#### 4.2.2 Kinetic Hydrate Inhibitor (KHI)

Low-dosage hydrate inhibitors (LDHIs) are generally classified into two main types: kinetic hydrate inhibitors (KHIs) and anti-agglomerants (AAs) based on the differentiated inhibition mechanism and the degree of subcooling (Δ*T*). KHIs are mostly amphiphilic polymers that contain polar amide groups and adjacent hydrophobic groups with short alkyl chains.^18,158^ In contrast to THIs, they do not alter the equilibrium conditions for hydrate formation, but they inhibit the onset of nucleation and reduce the rate of hydrates growth.^29,30,159^ Commercial KHIs include polymers synthesized from monomers such as *N*-vinylpyrrolidone (VP), *N*-isopropylmethacrylamide (NIPMAm) or *N*-vinyl caprolactam (VCap), as well as hyperbranched poly­(ester amide)­s (HPEAs).^21,160^ KHIs commonly used in industry are supplied as either liquids or solids, then diluted with a carrier solvent to the desired concentrations and introduced into the water phase of pipelines.

##### 4.2.2.1 Conventional KHIs

MD simulations have become a critical tool in predicting the performance of KHIs, providing insights into their mechanisms of action and aiding in the selection of the most effective inhibitors.^161^ *N*-Vinyl lactams homopolymers and copolymers, namely 5-ring *N*-vinylpyrrolidone (VP) and 7-ring *N*-vinyl caprolactam (VCap) are the most widely used commercial KHI formulations, whereas the 6-ring *N*-vinyl lactam monomer [*N*-vinyl peperidone (VPip)] is not commercially available as KHIs (Figure 9).^162^ MD simulations show that both PVP and PVCap monomers typically align at the hydrate-liquid interface. Both lactam groups can enter the open 5^12^6^4^ sII hydrate cavities. Their amide groups in the lactam ring form robust, double-bounded hydrogen bonds with interfacial water molecules, while the hydrophobic segment of the ring preferentially interact with hydrocarbons.^162^ This amphiphilic balance enables these polymers to reside at the interface where the polar groups disrupt the local water–water hydrogen-bond network, and the nonpolar parts interact with dissolved gases via van der Waal forces. Notably, PVCap exhibits much stronger attractive interactions with the hydrate than PVP, suggesting that the larger lactam ring size and enhanced hydrophobic part of PVCap improve its anchoring ability at the interface.^163^ This stronger attachment likely disrupts the local water structure more effectively, limiting the hydrogen-bond network formation for stable hydrate cages. In addition, PVCap is expected to be less soluble in water as compared to PVP, which promotes its retention at the surface, thereby further contributing to the inhibitory performance.^161^

**Figure 9:**
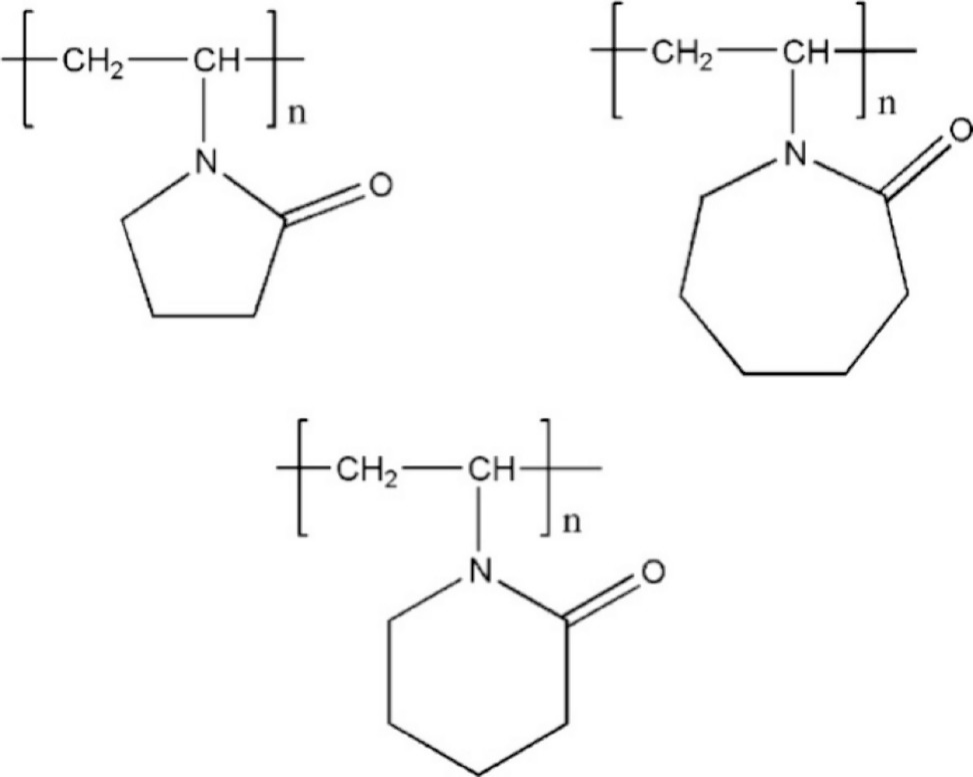
Structures of poly­(*N*-vinylpyrrolidone) (PVP) (top left), poly­(*N*-vinyl piperidone) (PVPip) (bottom), and poly­(*N*-vinyl caprolactam) (PVCap) (top right). This figure was reproduced with permission from ref 162. Copyright 2011 Elsevier.

Cheng et al. also studied the inhibitory effect of a series of copolymers, composed of *N*-vinylpyrrolidone and *N*-acrylate, on both CH_4_ hydrate and natural gas hydrate formation using molecular dynamics simulations.^164^ The results indicated that PVP-A, which introduced hydrophobic butyl ester group into PVP (*N*-vinylpyrrolidone) demonstrated the highest inhibitory capability (Figure 10). The longer alkyl chain in the ester group increases its hydrophobic interactions at the gas–liquid interface. This modification not only reinforces the anchoring of the inhibitor but also facilitates the formation of CH_4_ bubbles, thus restricting CH_4_ bubbles to redissolve into the liquid phase and participating in hydrate cage formation, (Figure 10). The findings suggest that the length of the alkyl chain in the ester group plays a crucial role, as longer chains further enhance the hydrophobic interactions that aggregate more CH_4_ molecules, strengthening the inhibitor’s effectiveness in gas hydrate management.^164^

**Figure 10:**
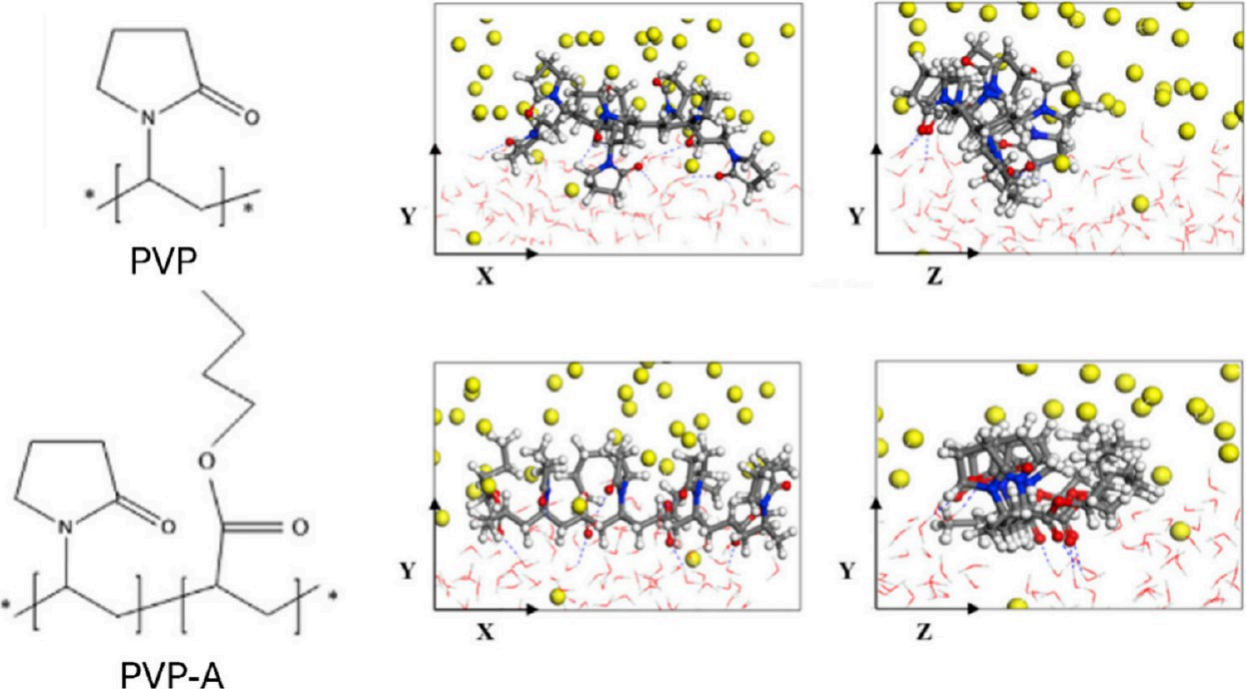
Locally enlarged snapshots of the systems with PVP (top) and PVP-A (bottom). This figur was adapted with permission from ref 164. Copyright 2022 KeAi/Elsevier. The inhibitor molecules are depicted using ball-and-stick models, with CH_4_ molecules shown as yellow spheres, and water molecules as lines. Within the inhibitor structure, oxygen atoms are colored red, hydrogen atoms white, carbon atoms gray, nitrogen atoms blue, while hydrogen bonds are illustrated as blue dashed lines.

In addition to conventional KHIs, there are chemicals with dual effects on gas hydrates, which are operationally and economically significant. For instance, Omidvar et al. introduced dual-purpose hydrate and corrosion inhibitors. These inhibitors incorporate active functional groups targeting both gas hydrate formation and corrosion in their chemical structure. They utilized maleic anhydride, a cost-effective monomer, to produce ionic *N*-vinyl caprolactam/maleic-based copolymers [P­(VCap-*co*-MA)] as promising dual-function inhibitors. MD simulations revealed that in the aqueous phase, the inhibitor’s cation forms a hydration shell which reduces the activity of water molecules and decreases methane solubility, lowering the driving force for hydrate nucleation. At the gas–liquid interface, the anion orients with its oxygen atoms toward the liquid to form hydrogen bonds while its hydrophobic alkyl groups face the gas phase, altering the interfacial tension and hindering methane migration. Furthermore, the VCap groups present in the anion enhance the water solubility of the inhibitor by forming additional hydrogen bonds with surrounding water, reinforcing these interfacial effects. On the hydrate surface, the anion embeds its alkyl chain and forms hydrogen bonds with water molecules, disrupting the local hydrogen-bond network and occupying methane adsorption sites. As the simulation proceeds, the cation also adsorb on the hydrate surface and embeds its ethyl group into the hydrate, contributing to a more significant inhibition.^165^ During the simultaneous injection of corrosion inhibitors and gas hydrate inhibitors in oil and gas pipelines, compatibility issues often arise, diminishing their effectiveness. Farhadian et al. developed dual-purpose inhibitors (DPIs) to address this challenge. Among these, DPI2, featuring a propyl pendant group, exhibited optimal performance, achieving a subcooling temperature of 18.1 °C at 5000 ppm concentration. Molecular dynamics (MD) simulations elucidated that the bulky anionic segment of the DPI2 molecule exhibits strong affinity for the hydrate surface, whereas its cationic counterpart predominantly resides in the surrounding solution. The orientation of the anionic part tends to be stretched along the hydrate surface. This adsorption partially covered the surface, acting as a barrier to mass transfer. Additionally, the inhibitor’s anion interacted with nearby water molecules, reducing water activity and facilitating the formation of hydrogen-bonding networks crucial for hydrate formation. Furthermore, the inhibitor demonstrated significant adsorption on the metal, forming a protective layer on steel surfaces.^166^ Hu et al. performed MD simulations to examine the impact of a conventional imidazoline corrosion inhibitor [1-(2-aminoethyl)-11-alkyl-imidazoline, AAI] on CH_4_ hydrate formation. The results found that its hydrophobic carbon tail (specifically the terminal C1 atom) forms a highly organized hydration shell with water, similar to that observed around methane molecules. However, fluctuations in the flexible carbon chain destabilize this hydrate-like arrangement, thereby retarding hydrate nucleation. As the concentration of AAI increases, the inhibitor aggregates into hydrophobic domains that attract methane to its hydrophobic region and form a methane nanobubble. The hydrophobic carbon chains of AAI exclude water molecules, effectively shielding methane from interactions with the surrounding aqueous environment. Meanwhile, the hydrophilic nitrogen atoms in the five-membered ring preferentially form hydrogen bonds with water, disrupting the water–water hydrogen-bond network. Although these hydrophilic interactions contribute to the inhibition, their effect is secondary to that of the hydrophobic domains, as a notable fraction of the hydrophilic moieties preferentially adsorb onto the iron surface rather than localizing in the hydrate-forming zone. Moreover, hydrates crystals were observed to form away from the inhibitor-covered iron surface. Free energy analysis of water cage migration toward the iron surface revealed that the inhibitor-coated pipeline surface discouraged gas hydrate deposition.^167^

Beyond conventional polymeric KHIs, recent studies have explored the potential of nanostructured carbon material as a novel kinetic inhibitor. A notable example is the study from He et al., which investigated the inhibitory effects of fullerene C_60_ on CH_4_ hydrate formation. The MD simulation results demonstrated that the inhibitory effects of C_60_ stem from its unique spherical structure and hydrophobic surface which repels water molecules, disrupting the formation of water clusters for hydrate formation. Additionally, C_60_ can absorb methane molecules on its surface through van der Waals interactions, leading to the localized aggregation of methane and thereby reducing their availability as guest molecules for hydrate formation. Due to its low thermal conductivity, C_60_ does not effectively support heat transfer during continuous hydrate formation. All these properties make it a potentially excellent kinetic hydrate inhibitor.^168^

##### 4.2.2.2 Environmentally Friendly Inhibitors

Although polymer-based KHIs demonstrate high performance, their limited biodegradability raises concerns about potential marine pollution.^18,169^ Additionally, their high viscosity and low solubility in water present significant challenges to broader applications.^170^ This underscores the shift toward more sustainable practices in managing hydrate formation in the oil and gas industry. Ionic liquids (ILs) are promising thermo-kinetic inhibitors for gas hydrate formation.^171,172^ These are electrolyte salts consisting of large organic cations paired with either organic or inorganic anions, resulting in compounds with low melting points. ILs are considered as environmental-friendly mainly because of their unique properties, which help reduce environmental impact compared to traditional solvents. For instance, ILs have low vapor pressures, significantly reducing the release of volatile organic compounds into the atmosphere. They are also highly stable and reusable, which minimizes waste and the need for frequent replacement. One of the most appealing features of ILs is the ability to tailor their functionalities, especially when paired with desirable traits like extremely low vapor pressure, thermally stable, non-combustible, and high dissolvent potential.^173^ The specific structural feathers of ILs, such as the presence and position of hydroxyl and oxygen groups and the length of the alkyl chains, enable them to form stable hydrogen-bond networks with interfacial water.^172^ These unique properties make them a potential alternative outperform conventional inhibitors.

A MD simulation study indicated that 1-ethyl-3-methylimidazolium chloride (EMIM-Cl) is the best inhibitor for CH_4_ hydrate formation as compared to other inhibitors like benzene, methanol, NaCl, and tetrahydrofuran (THF) within a carbon nanotube, highlighting the thermodynamic and kinetic advantages of using this ionic liquid.^174^ The inhibiting mechanism was explored by examining the evolution of the hydrate structure, revealing the joint action of the ability of ILs to hydrogen bonds and steric hindrance. As is known, a sI methane hydrate cell is composed of 5 ^12^ small cages and 5^12^6^2^ large cages, where each large cage consists of 12 five-membered rings and two six-membered rings located on opposite sides.^156^ Under the influence of EMIM, certain six-membered rings within the large cage structures developed from five-membered rings, partly hindering hydrate formation by perturbing the stability and symmetry of the hydrate lattice.^175^ The inhibition efficiency of six different imidazolium-based ILs were also investigated for CH_4_ hydrate formation. These compounds were chosen due to the variations in their anionic components and alkyl chain lengths on the imidazolium ring, which could potentially impact their inhibitory performance. In particular, the strong hydrogen bonds that form between these cations and water molecules, and consequently, the high thermodynamic inhibition, are caused by the presence of OH functional groups. Cations, more effective than anions in kinetic inhibition, tended to migrate in the direction of the hydrate crystal, form a bond with the hydrate surface, and keep water molecules from congregating near it.^176^ As for morpholinium ionic liquids, MD simulation results revealed that chloride anions (Cl^–^) act as kinetic hydrate promoters due to their ability to enhance the local structuring of water molecules, whereas tetrafluoroborate anions (BF_4_^–^) function as inhibitors, disrupting the hydrogen-bonding network of water.^177^ This illustrates the nuanced roles that different anions in ionic liquids can play in hydrate formation kinetics. The effects of quaternary ammonium and phosphonium ILs on CO_2_ hydrate formation were also studied based on the DFT and MD calculations. By comparing the interactions of anion–cation, anion–water, anion–CO_2_, and water–water in the designated systems, the results explained the main reason for the different role between the IL with its analogue counterpart, either as a hydrate promoter or inhibitor.^178^

Certain amino acids and their derivatives have also shown potential as kinetic hydrate inhibitors, offering a more biodegradable and less toxic alternative to the traditional KHIs. Amino acids, the building blocks of proteins, are usually made of an organic side chain, an amino group, and a carboxyl group.^179^ Similar to the above-mentioned additives, their side chain and hydropathy index play an important role in manipulating gas hydrate formation kinetics. The inhibitory performance is inversely correlated with the length of the alkyl side chain.^180,181^

According to a study from Oluwunmi et al., asparagine (Figure 11a) significantly impacted hydrate growth and successfully prevented CH_4_ hydrates formation during MD simulations. Instead of being dispersed throughout the bulk water, asparagine demonstrated a surface excess and was more hydrophilic than other tested KHIs that absorbed at the water/CH_4_ interface, thus leading to a higher efficiency in suppressing the growth of nanoclusters.^160^ It has also been established that serine (Figure 11b), glycine, alanine, and proline all inhibit the formation of CH_4_ hydrates, with serine acting as the most effective inhibitor among the candidates.^182^ The observed inhibition of water networks is primarily attributed to the mechanism by which amino acids form hydrogen bonds with water molecules. Moreover, serine, characterized by its hydrophilic properties, high solubility, a high number of donor and acceptor atoms, and the absence of a cyclic structure, emerges as the most effective inhibitor among other amino acids. This finding has also been supported by Hu et al.^183^ who investigated the effects of glycine (Figure 11c), serine and valine and demonstrated the highest adsorption energy and inhibition efficiency for serine. It should be mentioned that amino acids’ ability to function as promoters or inhibitors of various gas hydrates depends on their composition. In experimental investigations, glycine provided the highest inhibition for CO_2_ hydrates, followed by alanine (Figure 11d), valine (Figure 11e), leucine, and isoleucine, some of which promotes CH_4_ hydrate formation.^180^

**Figure 11:**
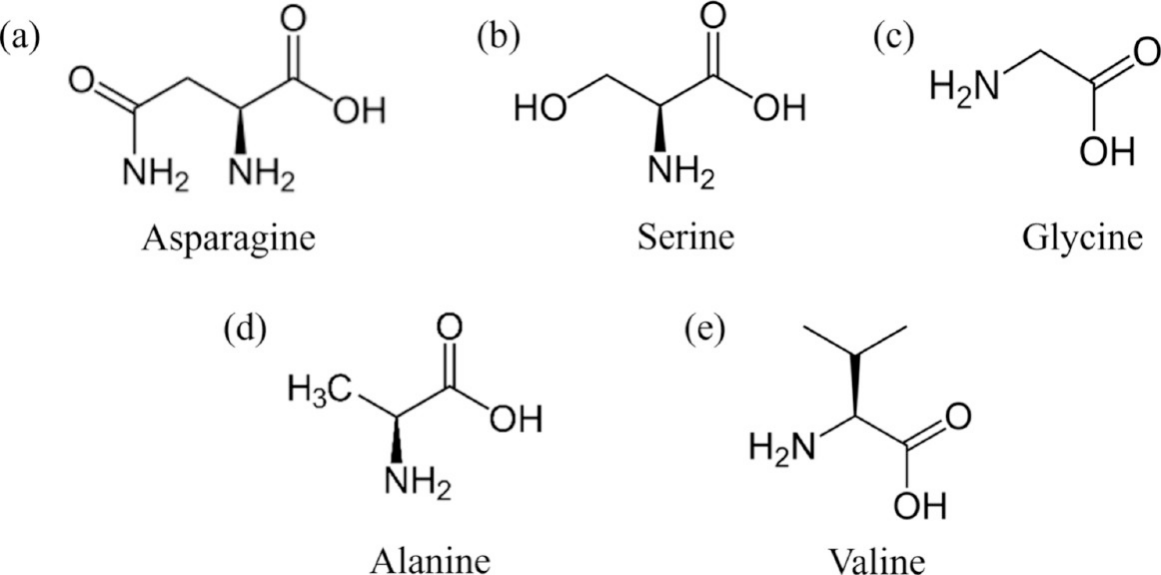
Structures of typical amino acids mentioned in the text as kinetic hydrate inhibitors: (a) asparagine, (b) serine, (c) glycine, (d) alanine, and (e) valine.

Research on amino acids has also led to advancements in studying peptides, chains of amino acids linked by peptide bonds, as KHIs. Three dipeptides have been proposed as effective CH_4_ hydrate inhibitors: alanine–alanine, alanine–glycine, and glycine–glycine dipeptides. Of these, alanine-glycine was the most successful. According to MD simulation results, hydrate nucleation and growth were impeded by the abundance of liquid-like water molecules and their more vigorous movements in the dipeptide-containing systems.^184^ Similar research on alanine-rich short peptides suggested that the additive adsorb at the hydrate-liquid interface, with the hydrophobic methyl groups docking into hydrate half-cages to immobilize peptides on the hydrate surface,^185,186^ as shown in Figure 12. The hydrate surface was coated with peptides that slowed down mass transfer between interfaces, which inhibited the subsequent growth of hydrates. Since the quantity of CH_4_ in the simulation box greatly surpasses the solubility, CH_4_ bubbles are also generated during the process (holes in Figure 12). Certain peptides stick to the bubble surface due to their amphiphilia, which keeps the gas–liquid interface stable.

**Figure 12:**
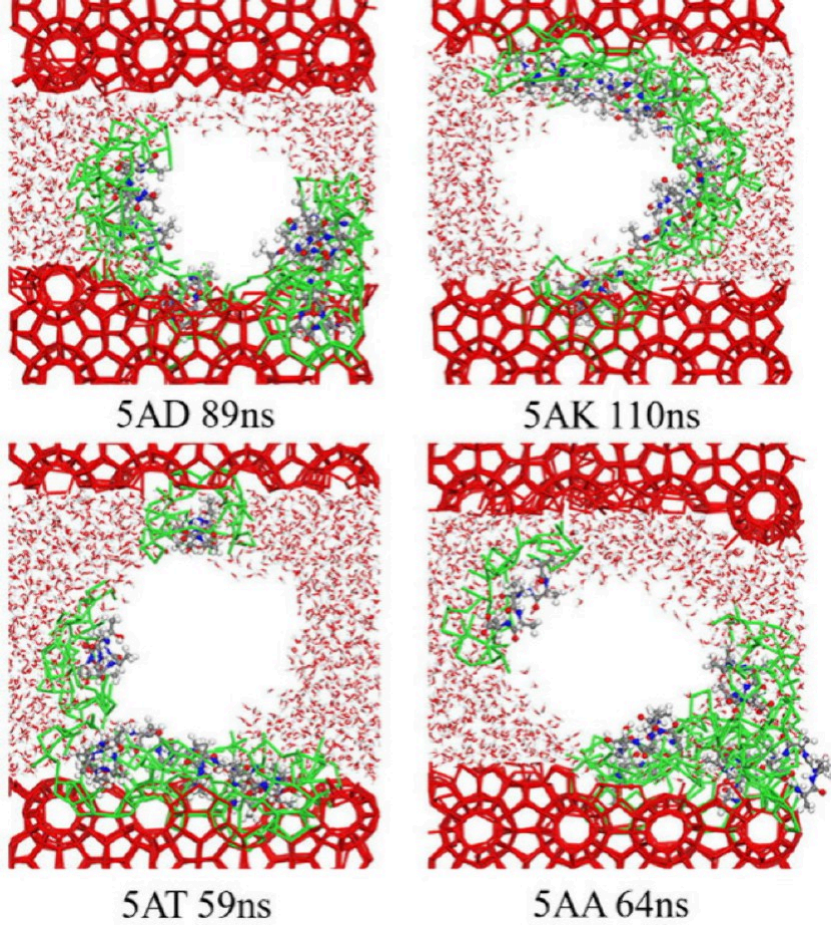
Snapshots of the moments when four different alanine-rich peptides just enter the water cages. This figure was adapted with permission from ref 186. Copyright 2023 Elsevier. The water cages around the peptides are highlighted in green for clarity.

The unique family of polypeptides known as antifreeze proteins (AFPs) is found in certain organisms (such as fish, plants, and insects) that survive in subzero temperatures. By adhering to the surface of ice crystals, AFPs prevent ice formation in a non-colligative way.^187^ The unique properties of AFPs have inspired research into their potential applications in gas hydrate inhibition. AFPs can be classified into five subtypes including types I, II, III, and IV and antifreeze glycoproteins according to their large vibrations in structure and activity,^188^ with type I a single straight helix, type II cysteine-rich globular AFPs and type III globular proteins devoid of any sequence repeat^189^ and type IV being predicted as an antiparallel helix bundle. AFPs, types I and III, have been experimentally confirmed to impede the formation of sI and sII gas hydrates in both demineralized and saline water systems.^190,191^ Maddah et al. modeled the formation of CH_4_ hydrate with AFP III and various mutations. It was discovered that the adsorption–inhibition mechanism of AFP III on hydrate and the antifreeze activity of AFP III is not directly attributed to the hydrogen bond formation. Furthermore, the findings proposed that hydrophobic interaction between AFP III and hydrate surface is dependent on the shape and geometry of the hydrate-binding surface of AFP, suggesting that the interface between hydrate and AFP is rather rigid.^192^ Later, they worked on Type I AFP and discovered that AFP induces curvature in hydrate growth patterns at the hydrate/water interface. Upon adsorption, the protein binds to the hydrate surface either from Thr2 or Thr35, the hydrate surface bends around residual 15–17 forming a curvature in hydrate surface.^193^ In line with the results of alanine-rich peptides previously reported, this also causes the methyl groups of Ala6, Ala18 and Ala20 to entrap in hydrate cavities at the interface.

#### 4.2.3 Anti-agglomerants (AAs)

Anti-agglomeration (AA) techniques are employed to prevent the formation of large clumps or masses of gas hydrate crystals, which can obstruct pipelines and hinder gas flow. These methods involve the use of additives or inhibitors to disrupt crystal growth or promote particle dispersion.^157^ AAs are surfactants that have a hydrophobic tail that stops particle aggregation and a hydrophilic headgroup that attaches to the surfaces of hydrate particles. It is widely accepted in the literature that AAs create water-in-oil emulsions, in which the water droplets subsequently convert into hydrate particles which cannot agglomerate.^157,194,195^ In essence, without the presence of the oil phase, there is currently no known mechanism for the anti-agglomeration process to occur.^156,196^

##### 4.2.3.1 Conventional AAs

Quaternary ammonium salts have emerged as highly effective anti-agglomeration (AA) agents, particularly at high supercooling temperatures. The surface adsorption of a quaternary ammonium salt anti-agglomerant inhibitor on a CH_4_–C_3_H_8_ sII hydrate surface in aqueous and liquid hydrocarbon phases was examined using MD simulations. The study of Bellucci et al. was able to effectively determine the best binding locations on the (111) crystal face of an sII CH_4_–C_3_H_8_ hydrate in their study. Additionally, they described the inhibitor’s equilibrium binding configurations and the associated binding energies. Their findings indicated that the inhibitor is less effective in the aqueous phase due to the less favorable surface adsorption, and they showed that the extent of surface adsorption is significantly greater in the liquid hydrocarbon phase compared to the aqueous phase. The *n*-dodecyl-tri­(*n*-butyl)­ammonium chloride, a quaternary ammonium (QA) molecule, demonstrated remarkable efficacy as an anti-agglomerant inhibitor in their simulations using a model anti-agglomerant. As anticipated, the QA molecule stabilizes the hydrate slurry created by the liquid hydrocarbon phase by adhering to the hydrate surface. By breaking the hydrogen bond networks between hydrate particles, AA may destabilize the capillary liquid bridges that form between them and stop them from aggregating, in addition to stabilizing the water-in-oil emulsion. As a result, their findings aligned with the pattern of experiments showing that AA works better in systems with an oil rather than a water predominance.^197^ In a different study, they looked into how NaCl affected the adsorption of a basic quaternary ammonium cationic surfactant molecule onto the CH_4_–C_3_H_8_ sII hydrate surface (see Figure 13).^198^ This work showed a synergistic effect of salts and QAs compared to previous work. When salt was added, the anti-agglomerant’s solubility in the solution decreased, which is why AA prefers to migrate to the hydrate’s surface area rather than the bulk. Anti-agglomeration relies heavily on adsorption to the hydrate surface. Furthermore, an interfacial layer that was negatively charged was created by the salt ions close to the hydrate surface. Because of the disruption of the hydrogen-bonding network of water near the hydrate interface caused by this layer, the AA molecule is stabilized in its bound state by the strong interaction of the chloride anions with its cationic head.^198^

**Figure 13:**
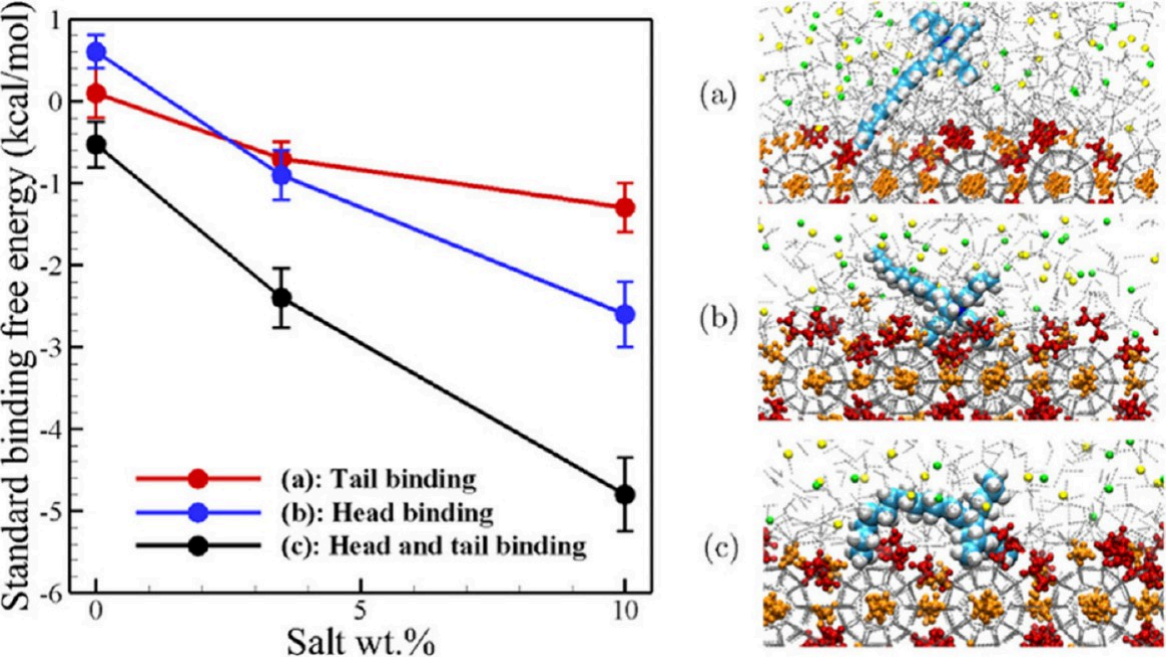
Standard binding free energy with salt concentration and AA molecule snapshots attached to the hydrate surface can be divided into three primary groups. (a) Only the AA molecule’s long hydrocarbon tail attaches to the hydrate surface. (b) AA molecule’s ionic head is the only part that binds to the hydrate surface. (c) Surface of the hydrate is bound by the AA molecule’s head and tail. This figure was reproduced with permission from ref 198. Copyright 2018 American Chemical Society.

A study examined the interactions between gas hydrate nanoparticles (sII CH_4_/C_2_H_6_ hydrates) in hydrocarbons (*n*-dodecane or *n*-heptane mixed with CH_4_/C_2_H_6_ gas) alongside various quaternary ammonium AAs (AAC8, AAC12, AAC121, and AAC171) under industrial conditions.^199^ The results of the simulation revealed important elements influencing AA performance, such as AA orientation at the hydrate-oil interface and alterations in entropy and free energy during solvation. Notably, molecular flexibility upon solvation emerged as crucial. If the repulsive peaks in the force–distance profiles are strong enough, AA can prevent coalescence, which is the purpose of using AA for hydrate antiaggregation. In particular, AAC8 performed better in *n*-heptane than in *n*-dodecane because of its higher cohesive force in *n*-dodecane and the fact that its molecules orient their long hydrocarbon tails more vertically in *n*-heptane. In contrast, AAC12, AAC121, and AAC171 showed opposing patterns. These insights offer valuable understanding for optimizing AA strategies in hydrate prevention.^199^

Mohr et al. conducted a study to rank the efficiency of AAs for sII hydrate inhibition using computational and experimental methods.^200^ They assessed AA performance by simulating the coalescence of a water droplet coated in surfactant molecules and a hydrate slab. The four AAs that were studied are all ammonium salts. The structure of the third and fourth AAs is simpler and lacks a spacer group, whereas the first two AAs are identical up to the counterion. However, instead of two alkyl tails, they have three. Additionally, the counterion for the third and fourth AAs (chloride) is much smaller compared to the counterions for the first two AAs. Four AAs revealed a well-ordered AA film on the hydrate surface. Results showed that both positively charged headgroups and chloride anions were close to the surface, with tail orientations transitioning from 45° to perpendicular with increasing concentration. This is significant because coalescence inhibition depends on interactions between the (covered) droplet and AA on the hydrate surface. A qualitative interaction between the droplet and the hydrate surface is provided by the force–distance profiles, particularly those of the initial repulsion. The minimum distance, where no coalescence occurred, is lowest for the fourth AA, so that at a distance smaller than this value, coalescence is inevitable. The study demonstrated good agreement between simulation predictions and experimental measurements, offering valuable insights into hydrate inhibition strategies.^200^ In the other work of this research group, the adsorption behavior of ten different hydrate AAs on a sII hydrate surface covered with varying thicknesses of liquid water layers was investigated.^201^ Despite having quaternary ammonium-based head groups, there were notable differences in molecular shapes. Additionally, the aliphatic tail chains varied in length. The research found that increased liquid water thickness on the hydrate surface led to less favorable adsorption of AAs, although individual molecules exhibited significant differences. The ionic AAs adsorb much better compared to the non-ionic ones. Additionally, the ideal liquid water layer thickness was found, which encourages the growth of hydrates because it contains both liquid water and guest molecules that form hydrates. On the other hand, because there were fewer guest molecules available close to the advancing hydrate front, thicker liquid water layers slowed the growth of hydrates.^201^

It has been also suggested that polycyclic aromatic compounds such as benzene, toluene, *p*-xylene, naphthalene, and pyrene can act as natural AAs.^202^ It should be noted that the use of the term “natural” here only indicates that these components occur in crude oil and does not indicate anything about the toxicity of these substances. Bui et al. investigated the potential of various aromatic compounds to act as natural AAs and their influence on synthetic AAs’ performance in preventing hydrate agglomerations by using MD simulations.^203^ Benzene and other monocyclic aromatics were found to break up surfactant films at low densities but to be ejected at high densities. In contrast, polycyclic aromatics, especially pyrene, stabilized surfactant films at both low and high density. This implies that polycyclic aromatics may improve certain surfactants’ performance whereas monocyclic aromatics may have the opposite effect. Polycyclic aromatics’ adsorbed layers effectively repel hydrate particles, suggesting that they can function as emulsifiers and AA. The results can be valuable for better understanding the synergistic and antagonistic effects related to stabilizing aqueous dispersions used in various applications.^203^

According to a number of studies, compounds based on carboxylic acids can also act as natural AAs.^202,204^ The effects of polynuclear aromatic carboxylic acids on gas hydrate particle agglomeration and the disruption of capillary liquid bridges between particles was explored.^205^ It identified two main AA processes: spontaneous surfactant adsorption onto hydrate surfaces and weakening of liquid bridges between attracted particles. MD simulations revealed that surfactant effectiveness depended on the intrinsic nature of their functional groups. Notably, 1-pyreneacetic acid demonstrated superior adsorption performance compared to other acids (2-naphthylacetic acid and 1-phenylacetic acid) due to its ability to alter structural preferences in aqueous solutions, enhancing hydrogen bond interactions with liquid bridges. The phenomenon that AAs delay hydrate growth by dispersing hydrate particles is similar to a KHI. Consequently, this kind of AA may be categorized as KHI-like.^205^ In another study from this group, it was demonstrated that the molecular structures of aromatic carboxylic acids can influence their adsorption behavior.^206^ Stronger interactions between acid molecules with more aromatic rings and the hydrocarbon phase cause the adsorption process to be slightly delayed. They can, however, considerably reduce interfacial tension. On the other hand, because of the strong π–π stacking interactions of the aromatic rings, acid molecules with more aromatic rings have a tendency to form stable aggregates in solution during the aqueous phase. Their adsorption to the hydrate/water interface is negatively impacted by this aggregation, which reduces their applicability at high water-cuts.^206^

However, quaternary ammonium salts and above-mentioned aromatic compounds were identified as highly effective anti-agglomeration (AA), their poor biodegradability and high toxicity pose significant challenges to use them in deep-water–oil and gas exploration. Consequently, there is a pressing need to develop environmentally friendly AA alternatives and enhance their inhibition activity. Such efforts would not only address environmental concerns but also offer a favorable green alternative, particularly in marine environments where sustainability is paramount.

##### 4.2.3.2 Environmentally Friendly AAs

An effective and environmentally friendly anti-agglomerant offers a viable way to reduce the possibility of gas hydrate blockages, with advantages for the environment and the economy.^207^ Biosurfactants, synthesized by microbial communities through the utilization of sugars and oils, are worth noting for their importance in diverse industries, most notably in oil recovery and bioremediation processes. They are recognized for their effectiveness in lowering surface tension and enhancing biodegradation processes. Biosurfactants are generally divided into four primary categories: glycolipids, fatty acids, lipopeptides, and polymers.^208^ They have garnered significant interest among researchers in recent years, owing to their notable characteristics, including excellent biodegradability, minimal toxicity, and exceptional stability across a wide range of temperatures, salinities, and pH levels.^209–211^

In the study by Tang et al., oleic acid was used to develop dual-functional inhibitors capable of both preventing corrosion and acting as anti-agglomerant hydrate inhibitors. The biobased anti-agglomerants (BAAs) effectively prevented gas hydrate agglomeration, maintaining constant torque during hydrate formation. MD simulations revealed that the headgroup of BAA1 adsorbs onto the hydrate surface, while its alkyl chain helps to disperse the formed hydrates within the hydrocarbon phase. As is typical for anti-agglomerants (AAs), BAAs form a stable emulsion in a water–paraffin mixture, preventing the hydrates from aggregating densely. As a result, the hydrates formed in BAA-containing solutions remained non-compact and could be easily detached from the stirrer bar. Electrochemical measurements demonstrated BAAs’ efficiency in inhibiting mild steel corrosion in simulated oilfield water containing H_2_S and CO_2_. BAA1 molecules adsorbed on steel surfaces provided optimal corrosion protection due to their parallel alignment.^212^ This highlights BAAs’ potential as multifunctional inhibitors in oilfield applications.^213^ In a subsequent study, Tang et al. synthesized a novel oleic acid derivative (OAD) and incorporated it into two eco-friendly, multifunctional agents designed to mitigate both hydrate agglomeration and corrosion in oil and gas pipelines. Experimental results demonstrated that the OADs significantly suppressed the growth of CH_4_ hydrates, leading to the formation of a flowable slurry within a water–paraffin system. MD simulations revealed that OAD adsorption decreases the hydrate surface hydrophilicity reducing hydrate and water droplet aggregation. The OADs also reduced binding energy between iron and corrosive species by 99.5%, forming a stable protective film on steel to resist acidic corrosion. The OADs modified the hydrate surface from hydrophilic to hydrophobic, as measured by water droplet contact angle, promoting CH_4_ hydrate dispersion and anti-agglomeration (see Figure 14). These findings underscore oleic acid’s potential as a sustainable source for developing dual-purpose hydrate and corrosion inhibitors.^214^

**Figure 14:**
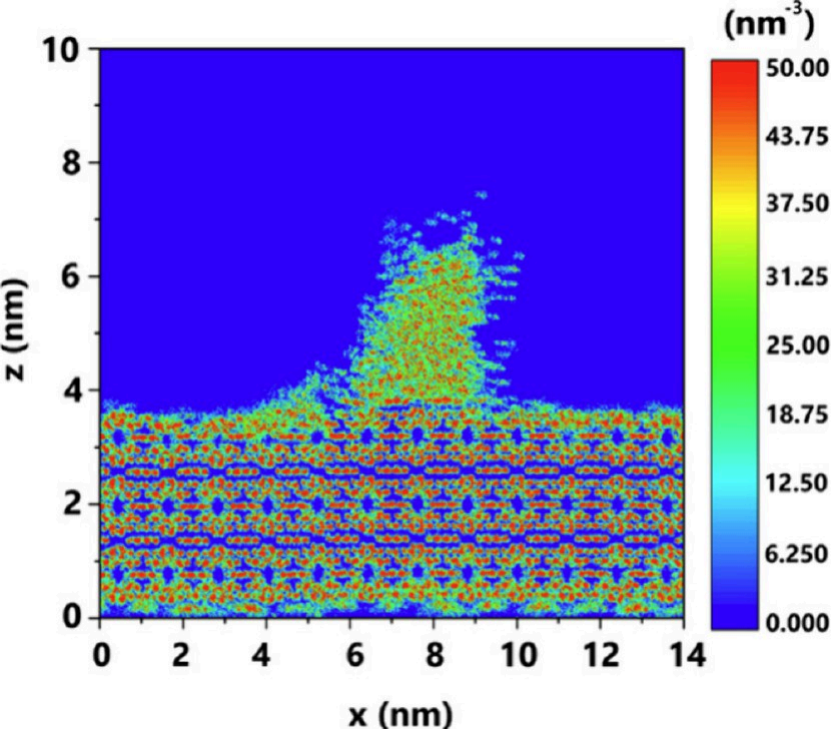
Density distribution of oxygen atom in water molecules after 100 ns within a system containing oleic acid derivative (OAD). This figure was adapted with permission from ref 214. Copyright 2024 American Chemical Society. Density distribution analysis revealed the boundary between the water droplets and methane hydrates. Without OADs, the water droplet’s contact angle on the hydrate surface was approximately 30°. With OAD added, the left and right contact angles increased to 58.8° and 68.7°, respectively, indicating a shift from hydrophilic to significantly more hydrophobic hydrate surface properties in the presence of OADs.

## 5 Challenges and Perspectives

While MD simulations provide valuable insights into molecular systems, their inherent time and size scale limitations present a significant challenge in aligning simulation outcomes with experimental observations. Experiments typically capture phenomena over longer time scales and larger systems than those currently accessible to MD simulations, making validation against experimental data difficult. In addition, simulating complex multiphase flows involving hydrates remains a significant challenge, requiring further research to develop effective solutions. Despite these challenges, MD simulations remain indispensable in scientific research, particularly in gas hydrate studies and flow assurance, due to their unique capabilities:

- Nucleation, growth, and dissociation insights: MD simulations allow researchers to investigate the nucleation, growth, and dissociation of gas hydrates on a molecular scale, offering insights that are challenging to obtain experimentally. This understanding is essential for developing effective strategies to manage hydrate formation and improve flow assurance in pipelines.
- Inhibitor assessment and design: MD simulations facilitate the evaluation of various inhibitors’ effectiveness in preventing hydrate formation, aiding in the development of more efficient and cost-effective chemical inhibitors. Conducting trial-and-error analyses solely through experimental methods can be expensive, time-consuming, and sometimes inconclusive. However, MD simulations allow scientists to predict and design new inhibitors by performing multiple simulation tests, selecting the most promising candidates, and then validating the results through targeted experimental testing. This approach significantly reduces both cost and time compared to traditional experimental methods.
- Risk assessment and prediction of unpredictable conditions: In real-world scenarios, numerous factors may be difficult to capture through experimental testing, and relevant data from the literature may be limited. In some cases, risks remain unclear and cannot be fully assessed through experiments alone. However, with a well-designed MD simulation, researchers can gain valuable insights at a lower cost compared to laboratory- or pilot-scale experiments. By simulating real conditions, MD simulations help scientists identify potential issues, understand their underlying causes, and develop effective strategies to mitigate them. Additionally, these strategies can first be tested in simulations before being implemented in real-world applications, reducing costs and uncertainties.

Consequently, to maximize the benefits of MD simulations while addressing their limitations, scientists can validate simulation results by comparing them with experimental data and try to fill the gap between molecular-scale simulations and macro-scale engineering applications. This qualitative comparison helps establish confidence in the simulation’s accuracy, particularly regarding time scales and system sizes, two critical factors in MD simulations. Beyond challenges related to time and system size, other critical factors, such as choosing suitable force fields for molecules and developing more reliable force fields for multicomponent systems, also significantly impact the accuracy of MD simulations. These challenges can be mitigated by carefully reviewing relevant literature and conducting trial MD simulations, allowing researchers to assess and refine the accuracy of their results.

In addition to the challenges posed by MD simulation limitations, there are still several research gaps in predicting the flow assurance of gas hydrates through MD simulations. While some valuable research has focused on risk assessment, there is a need to consider additional functional parameters that influence this process. This is also true for risk minimization strategies. For example, physical methods for managing gas hydrates, such as depressurization or heating, can sometimes be more cost-effective than chemical strategies. However, only a few studies have addressed this, with most focusing on chemical methods. Scientists can contribute more comprehensive research in this area, exploring various temperature and pressure conditions, as well as different hydrate structures, to better understand and improve flow assurance strategies.

There is a wide range of MD simulations focused on chemical inhibitors, designing new materials, and comparing them with traditional ones. However, there is still a significant gap in the development of green chemical inhibitors, which are crucial for environmental sustainability. Few research groups, such as Farhadian et al.,^30,214,215^ have made notable advancements in introducing green chemicals and evaluating them using highly accurate experimental methods. However, more attention from the scientific community is needed in this area, as it holds great importance for the future development of related fields.

It is also important to note that most methods developed through MD simulations or other computational techniques are research-focused and have yet to be introduced to policymakers or industry stakeholders. Scientists should strive to make their achievements more visible to a broader audience, including industry professionals, and explore opportunities for pilot testing. Additionally, statistical analysis is often lacking in such studies, with results presented more qualitatively. Incorporating statistical rigor would enhance the reliability and applicability of the findings.

## 6 Conclusion

The review addresses the challenge of gas hydrate formation in oil and gas pipelines, which disrupt operations and poses safety hazards. Strategies such as operational controls and chemical inhibitors are used to mitigate hydrate formation. Molecular dynamics (MD) simulations provide insights into hydrate behavior and intermolecular interactions, aiding in the development of better mitigation methods. The review focuses on MD simulations in flow assurance, emphasizing the need for ongoing research to improve the reliability, safety, and sustainability of hydrocarbon transportation systems.

Gas hydrates, along with other solids such as wax and asphaltene, pose significant threats to pipeline integrity and operational efficiency. Despite the interconnectedness of these solids in subsea flow systems, their interactions are often studied separately, leading to uncertainties in risk assessment. MD simulations offer a valuable tool for quantitatively evaluating hydrate blockage risk and understanding the role of oils in hydrate formation. Studies have investigated the precipitation of gas hydrates on solid surfaces, revealing the importance of surface properties in hydrate nucleation and adhesion. Furthermore, the influence of water content, pipe surface roughness, and hydrophobicity on hydrate stability has been examined, providing insights into hydrate evolution in pipeline systems. Moreover, the co-precipitation of gas hydrate with asphaltene and wax deposits has been explored through MD simulations. Asphaltenes at the water–gas interface promote hydrate formation, while wax molecules exhibit a dual role in either inhibiting or promoting hydrate growth depending on their distribution and size.

To address flow assurance challenges caused by gas hydrate formation in pipelines, the oil and gas industry employs some physical methods and chemical inhibitors. Physical strategies such as thermal stimulation, depressurization, and antigas hydrate surfaces offer various mechanisms to inhibit and mitigate gas hydrate formation. The rate and extent of hydrate dissociation depend on factors such as temperature, pressure, heating rate, and gas composition, as demonstrated in various MD simulations. The hydrate dissociation rate through depressurization is slower compared to thermal stimulation. Moreover, recent advancements in surface modification technology introduce the concept of antihydrate surfaces, which aim to prevent hydrate formation or facilitate the safe removal of hydrate plugs particularly in deep-water flow assurance. These surfaces leverage principles such as antinucleation, antideposition, and low adhesion to inhibit hydrate formation or deposition on pipeline surfaces.

Chemical inhibitors present a more economical and versatile approach compared to physical methods, offering targeted intervention points within the pipeline system. Thermodynamic hydrate inhibitors (THIs) such as alcohols, glycols, and salts modify the stability conditions of hydrate formation, hindering the nucleation and growth processes. However, conventional THIs like methanol and ethylene glycol, while effective, pose challenges in terms of high concentrations required and issues of recovery and recycling. In contrast, low-dosage chemical inhibitors (LDHIs) like kinetic hydrate inhibitors (KHIs) and anti-agglomerants (AAs) offer promising alternatives with lower concentrations needed and more sustainable profiles. KHIs, primarily composed of amphiphilic polymers, operate by delaying nucleation and slowing growth, thus preventing hydrate formation without affecting equilibrium conditions. Also, there are certain corrosion inhibitors that possess a dual effect, effectively targeting both corrosion and gas hydrate formation prevention. This capability significantly reduces operational costs for ensuring flow assurance in oil and gas pipelines. Despite their effectiveness, conventional KHIs exhibit limitations in terms of biodegradability and non-toxicity. Recent advancements have explored environmentally friendly alternatives such as ionic liquids (ILs) and amino acids derivatives, showcasing superior inhibition capabilities with reduced environmental impact. Anti-agglomerants (AAs) play a vital role in preventing the formation of large hydrate masses that can obstruct pipelines. Traditional AAs like quaternary ammonium salts are highly effective but suffer from biodegradability and toxicity concerns. Efforts to develop eco-friendly AAs have led to the exploration of carboxylic acid–based compounds and biosurfactants. These alternatives offer promising inhibition activity with reduced environmental impact, aligning with the growing emphasis on sustainability in the oil and gas industry. MD simulations were able to make important contributions to clarify the mechanisms of inhibition at a molecular level.

By using innovative strategies and materials, a combination of physical and chemical strategies, the industry can reduce the risks associated with gas hydrates while moving toward more sustainable methods of transportation and energy production. Further research and development in this field, supported by advanced simulation techniques such as MD, and addressing some of the key methodological aspects therein discussed and critiqued in section 2 (*vide supra*), is highly necessary to optimize existing simulation-supported flow-assurance strategies and to develop new solutions for efficient and safe hydrocarbon transportation systems.
